# Stress Tolerance-Related Genetic Traits of Fish Pathogen *Flavobacterium psychrophilum* in a Mature Biofilm

**DOI:** 10.3389/fmicb.2018.00018

**Published:** 2018-01-23

**Authors:** Héctor A. Levipan, Johan Quezada, Ruben Avendaño-Herrera

**Affiliations:** ^1^Laboratorio de Patología de Organismos Acuáticos y Biotecnología Acuícola, Facultad de Ciencias Biológicas, Universidad Andres Bello, Viña del Mar, Chile; ^2^Interdisciplinary Center for Aquaculture Research, Concepción, Chile; ^3^Centro de Investigación Marina Quintay (CIMARQ), Universidad Andrés Bello, Quintay, Chile

**Keywords:** *Flavobacterium psychrophilum*, fish pathogen, gene expression, RNA sequencing, planktonic cells, sessile cells, stress response

## Abstract

*Flavobacterium psychrophilum* is the causative agent of bacterial cold-water disease and rainbow trout fry syndrome, and hence this bacterium is placed among the most important salmonid pathogens in the freshwater aquaculture industry. Since bacteria in biofilms differ substantially from free-living counterparts, this study sought to find the main differences in gene expression between sessile and planktonic states of *F. psychrophilum* LM-02-Fp and NCMB1947^T^, with focus on stress-related changes in gene expression occurring during biofilm formation. To this end, biofilm and planktonic samples were analyzed by RNA sequencing to detect differentially expressed candidate genes (DECGs) between the two growth states, and decreasing the effects of interstrain variation by considering only genes with log_2_-fold changes ≤ −2 and ≥ 2 at *P*adj-values ≤ 0.001 as DECGs. Overall, 349 genes accounting for ~15% of total number of genes expressed in transcriptomes of *F. psychrophilum* LM-02-Fp and NCMB1947^T^ (*n* = 2327) were DECGs between biofilm and planktonic states. Approximately 83 and 81% of all up- and down-regulated candidate genes in mature biofilms, respectively, were assigned to at least one gene ontology term; these were primarily associated with the molecular function term “catalytic activity.” We detected a potential stress response in mature biofilms, characterized by a generalized down-regulation of DECGs with roles in the protein synthesis machinery (*n* = 63, primarily ribosomal proteins) and energy conservation (seven ATP synthase subunit genes), as well as an up-regulation of DECGs involved in DNA repair (*ruvC, recO, phrB1, smf*, and *dnaQ*) and oxidative stress response (cytochrome C peroxidase, probable peroxiredoxin, and a probable thioredoxin). These results support the idea of a strategic trade-off between growth-related processes and cell homeostasis to preserve biofilm structure and metabolic functioning. In addition, LDH-based cytotoxicity assays and an intraperitoneal challenge model for rainbow trout fry agreed with the transcriptomic evidence that the ability of *F. psychrophilum* to form biofilms could contribute to the virulence. Finally, the reported changes in gene expression, as induced by the plankton-to-biofilm transition, represent the first transcriptomic guideline to obtain insights into the *F. psychrophilum* biofilm lifestyle that could help understand the prevalence of this bacterium in aquaculture settings.

## Introduction

*Flavobacterium psychrophilum* is the infectious freshwater bacterium (Nilsen et al., 2011) responsible for bacterial cold-water disease (BCWD) and rainbow trout fry syndrome (RTFS) in aquaculture settings (Starliper, 2011). Additionally, this bacterium can infect a variety of non-salmonid freshwater fish (Sudheesh et al., 2012). Before the clinical onset of disease, *F. psychrophilum* can be detected by DNA-based real time-PCR in fish tank water rather than inlet water, being in turn undetectable through classical culture-based methods (Strepparava et al., 2014). Therefore, fish tanks can be an important risk factor (Madetoja et al., 2002) for bacterial replication and persistence in aquaculture settings, for instance, allowing biofilm establishment in different interfaces (e.g., solid-liquid, air-liquid). Consequently, when *F. psychrophilum* moves from a free-living state to a mature biofilm lifestyle, the bacterium may acquire emergent properties (Flemming et al., 2016) that favor persistence in an aquaculture setting, as well as infection recurrence (Sundell and Wiklund, 2011) in salmonids after biofilm-released cells return to the surrounding water. This idea is supported by a recent report on a coherent set of differentially expressed virulence- and biofilm-related genes between sessile and free-living cells, with most of these genes being significantly up-regulated in mature *F. psychrophilum* biofilms (Levipan and Avendaño-Herrera, 2017).

However, the study of mature bacterial biofilms is technically complex. For example, RNA degradation is a major barrier for gene expression analysis in biofilm cells using certain RNA-based approaches, such as RNA sequencing (RNA-seq). This is due to that mature biofilms are mainly composed of cells over the entire range of physiological states, from highly active to dormant and even dead (Flemming and Wingender, 2010; Flemming et al., 2016). The wide spectrum of metabolic states in biofilm cells is probably due to the influence of diverse stressor agents that mature biofilms often experience, thereby contributing to the development of enhanced stress resistance among sessile cells (Lee et al., 2014). Thus, conclusions derived from experimental designs that ensure optimal RNA-sample quality may not be representative of *in-situ* properties of some biological systems (Romero et al., 2014) such as bacterial mature biofilms. Nonetheless, because of the high sensitivity in transcript detection and a relatively low cost, RNA-seq technologies have been progressively adopted in biofilm research (Tan et al., 2015; Yan et al., 2017), providing high-quality data for analysis of gene-gene interactions based on noncoding RNAs, protein-coding genes, and even novel transcriptionally active sites (Nobile et al., 2012; Castro et al., 2017).

Interestingly, no stress-related gene expression pattern in *F. psychrophilum* biofilms has been reported hitherto, which in turn may not be easily discernible solely from the study of sessile cells. The present study identified 349 differentially expressed candidate genes (DECGs) between biofilm and planktonic growth states of *F. psychrophilum* LM-02-Fp and NCMB1947^T^ using a RNA-seq approach. A potential stress-resistance pattern in mature *F. psychrophilum* biofilms was found, as characterized by an important down-regulation of a gene repertory encoding for ribosomal proteins, transcriptional factors, different subunits of DNA-directed RNA polymerase, translation factors, elongation factors, electron carriers, and subunits of ATP synthase. In addition, an important up-regulation of genes responsible for DNA repair and some genes associated with the oxidative stress response was detected in sessile cells. Finally, the obtained results represent an initial baseline for future research on the biofilm lifestyle in *F. psychrophilum*.

## Materials and methods

### Bacterial strains and growth conditions

Strains LM-02-Fp and NCMB1947^T^ were confirmed as *F. psychrophilum* through standard phenotyping and 16S rDNA-based PCR as previously described (Urdaci et al., 1998; Bernardet et al., 2002). The two strains were routinely cultured with agitation (150 rpm) at 17 ± 1°C in half-strength TYES medium (Holt et al., 1993), and with not more than two subcultures grown from glycerol-amended stock cultures (15%, final concentration) and stored at −80°C. Briefly, 6-well microtiter plates (flat-bottom TPPR plates, Switzerland) containing a 5-cm^2^ sterile glass slide in every well were inoculated with 1.8 mL of culture to achieve an average density per strain of 1.77 ± 0.46 × 10^7^ CFU cm^−2^ (±SD). Recently, it has been demonstrated experimentally that maximum biofilm formation in these two strains occurs between 96 and 120 h (Levipan and Avendaño-Herrera, 2017) and hence, mature biofilms were allowed to form on glass slides with agitation (40 rpm) at 17 ± 1°C for 96 h. Afterward, 12 glass slides were collected per strain and washed by immersion in chilled sterile milli-Q water (10 s) for subsequent RNA extraction from biofilm cells. These samples were compared with 48-h-old planktonic samples (i.e., late exponential growth stage), since we could not obtain RNA extracts from 96-h-old biofilm supernatants with a RNA integrity number (RIN) adequate for RNA-seq. Planktonic cells from 48-h-old cultures of *F. psychrophilum* LM-02-Fp and NCMB1947^T^ were harvested by centrifugation (5780 RCF) at 4°C for 2.5 min for RNA isolation.

### RNA extraction and RT-qPCR

Total RNA was extracted with the TRIzol® Max™ Bacterial RNA Isolation Kit (Ambion, Thermo Fisher Scientific, NY, USA) from planktonic and biofilm samples of *F. psychrophilum* LM-02-Fp and NCMB1947^T^. To do so, planktonic cell pellets and biofilms formed on glass slides were incubated with 1 mL of TRIzol reagent for cell lysis, according to the manufacturer's specifications. Concentrations and preliminary qualities (A_260_/A_280_ ratio) of extracts were determined using a Nanodrop® ND-1000 spectrophotometer (Thermo Fisher Scientific) and formaldehyde-agarose gel electrophoresis (Rosen and Villa-Komaroff, 1990). Total RNA from four independent experiments was pooled per strain and growth state (biofilm vs. planktonic) at the same final concentration. The RNA integrity numbers of the four resultant RNA pools were determined on an Agilent Bioanalyzer using the RNA 6000 Nano Kit (Agilent Technologies, CA, USA). Afterward, each pool of total RNA was treated with the TURBO DNA-free™ Kit (Applied Biosystems, Austin, TX, USA) to be used as a RNA template for subsequent RNA-seq experiments.

Nine DECGs in RNA-seq data (see below) were randomly selected for designing qPCR primers (Supplementary Table 1) using the software Primer3 (version 4.0.0, Rozen and Skaletsky, 2000) and Beacon Designer™ (free edition). Reverse transcription-qPCR (RT-qPCR) assays were conducted with TURBO-treated RNA samples obtained from three independent experiments and following the procedures described elsewhere (Levipan and Avendaño-Herrera, 2017) with two modifications. (1) The complementary DNA (cDNA) was synthetized using random primers from the ImProm-II™ Reverse Transcription System (Promega, Madison, CA, USA). (2) The qPCR program consisted of an initial denaturation (3 min at 95°C) followed by 40 amplification cycles consisting of denaturation at 95°C for 30 s, 1 min annealing at a temperature chosen based on the primer pair to be used (Supplementary Table 1), and 1 min extension at 72°C. The efficiencies and correlation coefficients of standard curves for DECG quantification were 97.99 ± 2.86% and 0.9975 ± 0.0022 (mean ± SD), respectively. qPCR assays were carried out on a Stratagene Mx3000P real-time PCR device (Agilent Technologies-Stratagene) and data were processed with the software MXPro (version 4.10; Agilent Technologies).

### RNA-seq and data processing

Next-generation sequencing libraries were constructed in the AUSTRAL-omics Laboratory, Universidad Austral de Chile (Valdivia, Chile). Efficient rRNA depletion from each pool of total RNA was only achieved once the probes of three hybridization-based kits (i.e., RiboMinus™ Transcriptome Isolation Kit; Ribo-Zero™ rRNA Removal Kit; and MICROBExpress™ Bacterial mRNA Enrichment Kit) were mixed and used in two rounds of depletion, as per the MICROBExpress™ Kit protocol (Ambion, Thermo Fisher Scientific, NY, USA). Afterward, 16S- and 23S-rRNA-depleted samples were processed for the removal of ssRNAs < 100 nt and dsRNAs < 200 bp using the MEGAclear™ Kit (Ambion). An Agilent Bioanalyzer profile of rRNA-depleted samples confirmed the efficient removal of 16S- and 23S-rRNAs, as well as of small RNAs. Briefly, libraries were generated with the KAPA Stranded mRNA-seq Kit (Kapa Biosystems Inc., MA, USA) and labeled with a barcode for pooled-library sequencing. Library size determinations were performed by running the libraries on a Fragment Analyzer™ System (Advanced Analytical Technologies, IA, USA) and using the DNF-479 Standard Sensitivity NGS Fragment Analysis Kit. Library concentrations were determined on a LightCycler® Nano Real-Time PCR instrument (Roche Diagnostics, GmbH) and using the KAPA Library Quantification Kit (Kapa Biosystems, Inc.) to adjust concentrations to 10 nM. Libraries were pooled in an equimolar ratio using a protocol for denaturing and diluting libraries from the MiSeq System. The pooled libraries were then loaded (at 12 pM with 1% PhiX, final concentration) on an Illumina MiSeq instrument (Illumina, CA, USA) to create paired-end reads (2 × 150 bp) by using MiSeq Chemistry v2.

Technical sequence removal and quality trimming were performed with Trimmomatic (Bolger et al., 2014) and PRINSEQ (Schmieder and Edwards, 2011), respectively. Bases (or sequences) with low quality Phred scores (Phred < 30) were discarded. Genes in the reference genome for *F. psychrophilum* strain JIP02/86 (NCBI accession number NC_009613.3) were extracted using the “gffread” option in BedTools (Quinlan and Hall, 2010), thus generating a transcript FASTA file with all gene coordinates in the genome. The “.bam” files were obtained after mapping high-quality reads against extracted genes using the Burrows-Wheeler Alignment Tool (Li and Durbin, 2009). The number of sequence reads per gene for each “.bam” file was computed using the FASTA file of transcripts and the “multiBamCov” option in BedTools. High-quality reads were functionally annotated using the Blast2GO software (Conesa et al., 2005) based on BLAST algorithm run against the non-redundant (NR) database at NCBI and gene ontology (GO) for the hit sequences. The resultant GO terms for each gene were mapped against a total of 124 Go-Slim categories (Hu et al., 2008) in three major biological ontologies: biological processes, molecular functions, and cellular components.

RNA-seq data were deposited in the European Nucleotide Archive (ENA) under the PRJEB14670 accession number for planktonic (ERS1231641, ERS1231642) and biofilm (ERS1231643, ERS1231644) samples. The sequencing data of each sample are shown in Supplementary Table 2.

### Virulence testing

Chinook CHSE-214 cells (ATCC 1681) were grown into 24-well cell culture plates (5 × 10^4^ cells per well) using the Leibovitz's L-15 medium (HyClone Laboratories, Inc., Logan, Utah) supplemented with: 10% fetal bovine serum (Gibco, Invitrogen Laboratories), 6 mM L-glutamine (Gibco), 15 mM HEPES pH 7.3 (Gibco), and 100 μg mL^−1^/100 IU mL^−1^ streptomycin/penicillin (Gibco). Salmon embryo cells were incubated at 18°C and grown to 70–80% confluence. Before bacterial infection assays, chinook cells were washed with phosphate buffered saline (1X PBS, pH 7.0) and 2 mL of fresh antibiotic-free medium supplemented with 2% fetal bovine serum (Gibco) were added per well. The infection was performed with biofilm and planktonic cells of *F. psychrophilum* LM-02-Fp and NCMB1947^T^ grown as previously described. Briefly, planktonic cells were harvested from supernatants surrounding mature biofilms by centrifugation (5780 RCF) at 4°C for 2.5 min. Planktonic pellets were washed once with chilled sterile milli-Q water and resuspended in the same solvent at 5.80 ± 2.82 and 4.03 ± 2.70 × 10^4^ CFU mL^−1^ (mean ± SD) for inoculation with LM-02-Fp and NCMB1947^T^ strains, respectively. Glass slides with 96-h-old biofilms were washed by immersion (10 s) in chilled sterile milli-Q water before cell detachment with sterile cell scrapers. Biofilm-detached cells were concentrated in chilled sterile milli-Q water at 6.56 ± 2.22 and 3.35 ± 2.71 × 10^4^ CFU mL^−1^ (mean ± SD) for infection with LM-02-Fp and NCMB1947^T^ strains, respectively. One hundred microliters of planktonic and biofilm inocula were separately added to wells with CHSE-214 monolayers to study cytotoxic effects of *F. psychrophilum* in three independent experiments performed in triplicate at 18°C for 58 h. In addition, to evaluate the background cytotoxicity (BC) due to aging effects of the medium caused by bacterial growth, every experiment included CHSE-214 monolayers inoculated in triplicate with 100 μL (at 1.0 ± 0.72 × 10^4^ CFU mL^−1^) of the exponentially growing non-pathogenic *Escherichia coli* Stbl2. Cytotoxic effects induced by *F. psychrophilum* and *E. coli* (including CFU counts) were measured at 0, 10, 24, 34, and 58 h post-infection (hpi) using 100 μL-aliquots of cell culture supernatants and the lactate dehydrogenase (LDH) cytotoxicity detection kit (Takara Bio Inc., Otsu, Japan), in accordance with the manufacturer's instructions. This kit allows for the colorimetric detection of LDH activity-based cytotoxicity in cell-free supernatants by spectrophotometric reading at 500 nm (Tecan Microplate Reader, Infinite 200 PRO, Männedorf, Switzerland). Absorbance measurements were corrected for the background in low controls (i.e., chinook cells incubated without bacteria) and expressed as a percentage of the LDH activity in high controls (i.e., chinook cells incubated without bacteria with 1% Triton X-100) using the equation provided by the manufacturer. Cytotoxicity percentages induced by sessile and planktonic cells of *F. psychrophilum* were corrected for the average percentage of the BC determined with the Stbl2 strain, which was about 10%. Thus, we assumed that the growth of *F. psychrophilum* itself was not usually associated with higher cytotoxicities than this threshold percentage. In fact, *E. coli* Stbl2 had a faster growth in the cell culture medium (from 6.0 ± 3.46 × 10^2^ to 1.1 ± 0.61 × 10^7^ CFU mL^−1^ within 58 hpi) than *F. psychrophilum*, for example, compared with LM-02-Fp biofilm cells (from 1.4 ± 0.51 × 10^3^ to 2.3 ± 0.85 × 10^7^ CFU mL^−1^ within 58 hpi), but did not induce evident degenerative damage in the CHSE-214 cell line after 58 hpi (Supplementary Figure 1).

Also, planktonic and biofilm cells of *F. psychrophilum* LM-02-Fp and NCMB1947^T^ were evaluated for their virulence capacity on rainbow trout fry (*Oncorhynchus mykiss*) using an intraperitoneal challenge model for 26 days. Before bacterial challenge, fish were anesthetized by immersion in a benzocaine solution (30 mg L^−1^) at 18°C. In addition, the absence of *F. psychrophilum* or other bacterial pathogens was previously confirmed by bacteriological and PCR analyses of samples (gills, mucus, skin, spleen, and kidney) from randomly collected fish (Urdaci et al., 1998; Bernardet et al., 2002). Fish (5–8 g) were deposited and acclimatized (for 1 week) into 10 L tanks with aerated and dechlorinated water (7 L) to get an average density of 10.2 kg m^−3^ (11 fish per tank). In total, two tanks were used for each tested growth state and strain, including control tanks. Glass slides with 96-h-old biofilms of *F. psychrophilum* LM-02-Fp and NCMB1947^T^ were washed by immersion in chilled sterile milli-Q water for 10 s, detached as previously described, and resuspended in the same solvent at 5.13 ± 1.33 and 1.21 ± 0.2 × 10^4^ CFU mL^−1^ (mean ± SD), respectively. Similarly, planktonic cells of LM-02-Fp and NCMB1947^T^ strains were harvested and washed as previously described, and resuspended in chilled sterile milli-Q water at 4.34 ± 2.52 and 1.58 ± 0.53 × 10^4^ CFU mL^−1^, respectively. One hundred microliters of biofilm and planktonic inocula were intraperitoneally injected per fish, while fish in control tanks were injected with only 100 μL of sterile milli-Q water. Tanks were kept in a climatized room at 17 ± 1°C using a 12 h: 12 h light: dark regime. Tank water (pH 7.6–7.8) was changed bi-daily and fish were fed daily at 1.5% body weight. Dead fish were collected and analyzed by direct streaking of samples (kidney, liver, and spleen) onto TYES agar plates incubated at 18°C for 5 days. Biochemical and PCR analyses of isolates were performed to confirm whether the observed fish mortality in the experimental tanks was caused by *F. psychrophilum* in comparison with euthanized control fish.

### Data analysis

Since full transcriptomes of *F. psychrophilum* NCMB1947^T^ and LM-02-Fp differed significantly between biofilm and planktonic growth states, but not among the two strains (Levipan and Avendaño-Herrera, 2017), the current study reports DECGs between growth states that were shared by both *F. psychrophilum* strains. For this purpose, read counts derived from biofilm and planktonic states were separately normalized using TMM normalization in the edgeR Bioconductor Package (Robinson et al., 2010). The normalized reads were then simultaneously analyzed with DEseq2 to obtain a fold-change (FC) of gene expression (Love et al., 2014). FC thresholds for up- and down-regulated candidate genes in mature biofilms were 4- (log_2_-FC ≥ 2) and 0.25-fold (log_2_-FC ≤ −2), respectively, at a *P*adj-value of ≤0.001. In addition, to validate global shifts in gene expression obtained from RNA-seq data, nine DECGs (Supplementary Table 1) were randomly chosen for RT-qPCR assays using samples collected from three independent biofilm experiments. RT-qPCR data were normalized by the concentration of total RNA in planktonic and sessile samples and then log_2_-transformed before FC ratio computation between biofilm and planktonic states. FC thresholds for RT-qPCR data analysis were the same used in RNA-seq data analysis to identify DECGs between biofilm and planktonic states.

The LDH-based cytotoxicity of *F. psychrophilum* NCMB1947^T^ and LM-02-Fp on CHSE-214 cells was modeled in response to changes in predictor variables such as the growth state (biofilm vs. plankton), temporality, strain, and the interaction among the first two variables. This was done using generalized additive models for location, scale and shape from R “gamlss” package (Rigby and Stasinopoulos, 2005). Before run the analysis, the temporality was scaled (Z-transformed) in order to determinate its effect on the rest of variables (Schielzeth, 2010). The two strains were modeled together and separately using the beta inflated distribution (BEINF) with the time as a continuous variable. The BEINF is similar to the beta distribution but allows zeros and ones as values for the response variable (Ospina and Ferrari, 2010). Gehan-Breslow-Wilcoxon and Log-rank (Mantel-Cox) tests were performed with GraphPad Prism 7 (GraphPad Software, Inc., CA, USA) to determine statistically significant differences (*P* < 0.05) among mortality curves constructed from fish challenge data.

## Results

### Differentially expressed candidate genes (DECGs) between biofilm and planktonic states of *F. psychrophilum* LM-02-Fp and NCMB1947^T^

**Figure 1:**
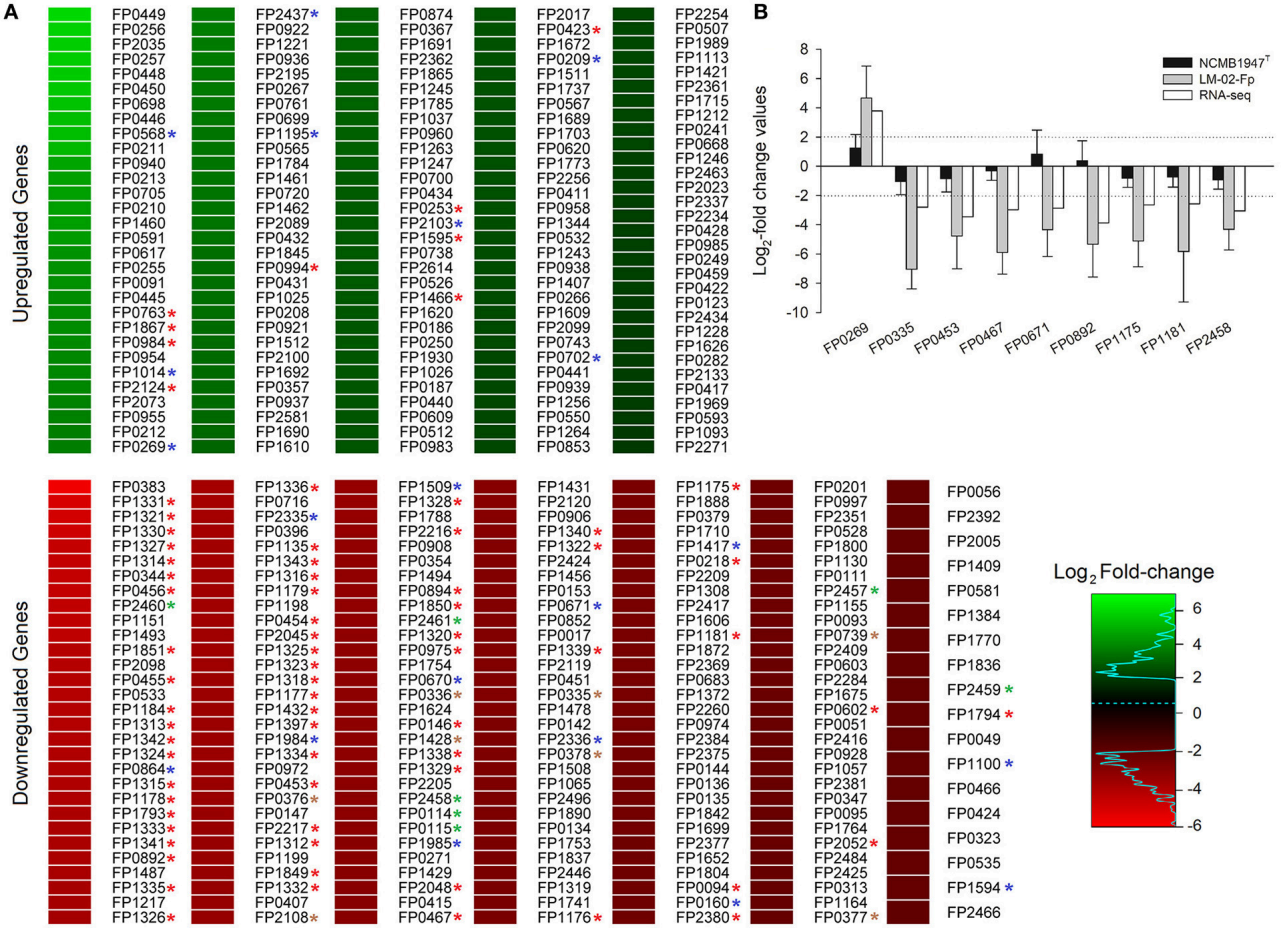
Total number of DECGs between biofilm and planktonic states of *F. psychrophilum* LM-02-Fp and NCMB1947^T^. **(A)** DECGs are indicated by locus tags according to the annotations for the *F. psychrophilum* JIP02/86 reference genome. A full description of every DECG is provided in Table 1 and Supplementary Table 3. DECGs potentially involved in adaptive stress response of biofilms are indicated by color asterisks (red, protein synthesis machinery; blue, DNA repair and antioxidant activity; brown, electron transport; green, ATP synthesis). **(B)** RNA-seq-derived fold change (FC) values are compared with RT-qPCR-based FC values for nine randomly selected DECGs. RT-qPCR data are averages ± SD of three independent experiments. Log_2_-FC values ≥ 2 and ≤ −2 in **(A,B)** indicate up- and down-regulated genes in the mature biofilm state, respectively.

**Figure 2:**
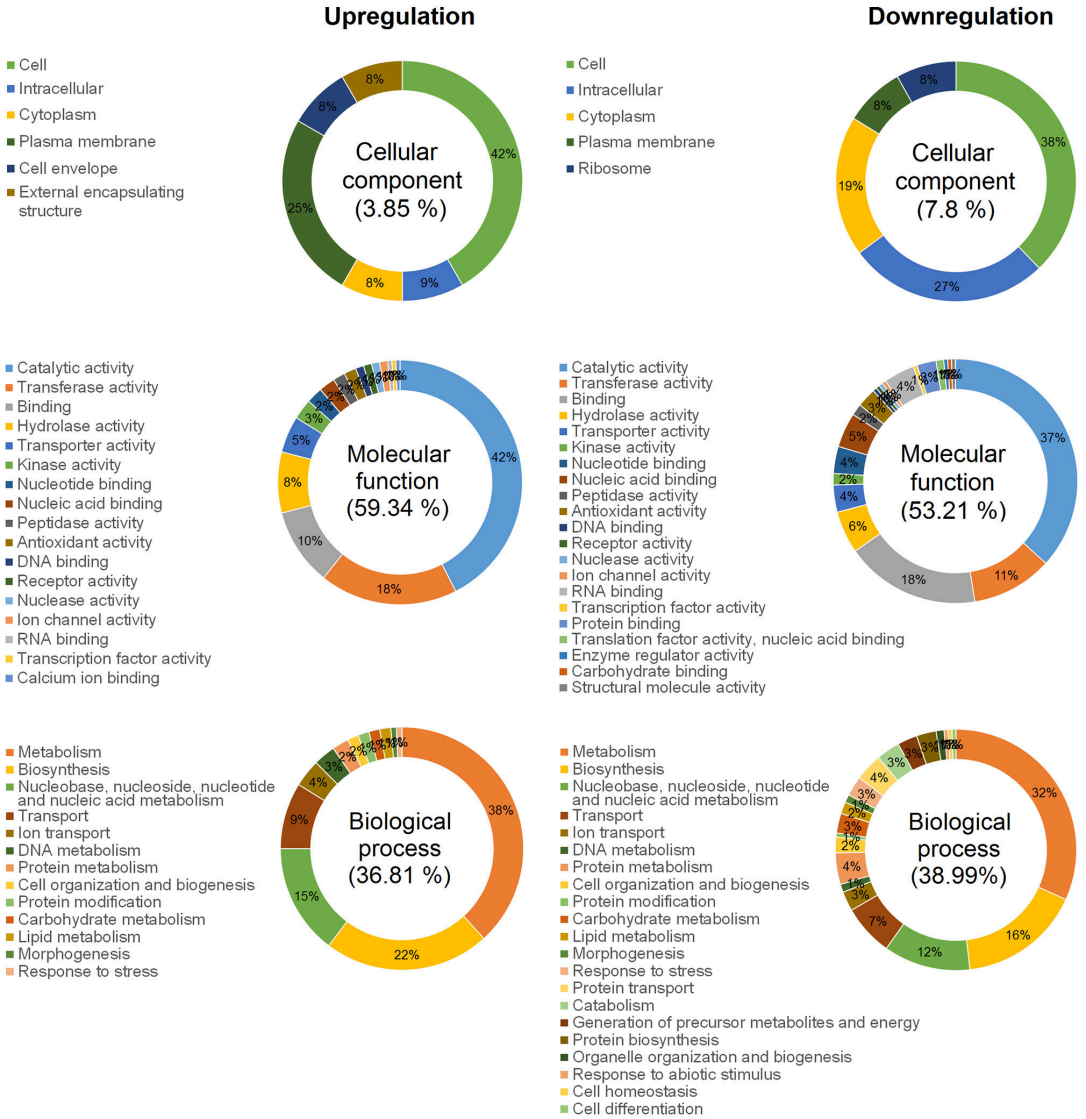
Ontology analysis of total DECGs between biofilm and planktonic states of *F. psychrophilum* LM-02-Fp and NCMB1947^T^. Pie plots show GO term occurrences summarized in specific Go-Slim categories after mapping unique GO terms against 124 Go-Slim categories associated with top-level ontologies for cellular components, molecular functions, and biological processes.

We identified 349 DECGs between biofilm and planktonic states, which accounted for ~15% of total number of genes expressed in transcriptomes of the two strains (i.e., 2327 expressed genes). Out of 349 DECGs, 151 and 198 were significantly up- and down- regulated in the mature biofilm state of both strains, respectively (Figure 1). Some 83% of all up-regulated candidate genes were associated with at least one GO term, resulting in a total of 392 term occurrences (Table 1 and Supplementary Table 3). Among these, 182 unique GO terms were grouped in different GO_Slim categories into biological processes (36.81%), molecular functions (59.34%), and cellular components (3.85%) (Figure 2). Similarly, 81% of all down-regulated candidate genes were associated with at least one GO term, resulting in a total of 591 term occurrences (Table 1 and Supplementary Table 3). The identified 218 unique GO terms were grouped in different Go-Slim categories into biological processes (38.99%), molecular functions (53.21%), and cellular components (7.8%) (Figure 2).

**Table 1:** Changes in gene expression as part of the potential adaptive response of *F. psychrophilum* biofilms (NCMB1947^T^ and LM-02-Fp) to face stressful conditions.

**Category**	Locus tag^*^	**DECG description**	Hit name^**^	**GO number**	Log_2_ FC^***^
Protein synthesis machinery	FP0094	23S rRNA-intervening sequence protein	FPSM_00097	GO:0005840	−2.38
	FP0146	50S ribosomal protein L25/general stress protein Ctc	*rplY*	GO:0005840, GO:0003735, GO:0008097, GO:0006412	−3.13
	FP0218	Molecular chaperone	FPSM_00244, *dksA*	GO:0008270	−2.63
	FP0253	Probable transcriptional regulator, AraC family	FP0253, FPSM_00280	GO:0003700, GO:0043565, GO:0006351, GO:0006355	2.82
	FP0344	30S ribosomal protein S16	*rpsP*	GO:0005840, GO:0003735, GO:0006412	−4.52
	FP0423	Probable transcriptional regulator, TetR family	FPSM_00466, FP0423	GO:0003677, GO:0006351, GO:0006355	2.64
	FP0453	Elongation factor Ts (EF-Ts)	*tsf*	GO:0005737, GO:0003746, GO:0006414	−3.45
	FP0454	30S ribosomal protein S2	*rpsB*	GO:0015935, GO:0003735, GO:0006412	−3.62
	FP0455	30S ribosomal protein S9	*rpsI*	GO:0005840, GO:0003735, GO:0006412	−4.15
	FP0456	50S ribosomal protein L13	*rplM*	GO:0005840, GO:0003735, GO:0006412	−4.51
	FP0467	Universal stress protein UspA	*uspA*, FPSM_00512, FP0467	GO:0006950	−2.98
	FP0602	RsfS-iojap-like ribosome-associated protein (ribosomal silencing factor)	FP0602, *rsfS*	GO:0005737, GO:0017148, GO:0042256, GO:0090071	−2.26
	FP0763	Probable transcriptional regulator, LysR family	FPSM_01554, FP0763	GO:0003677, GO:0003700, GO:0006351, GO:0006355	4.12
	FP0892	50S ribosomal protein L20	*rplT*	GO:0005840, GO:0003735, GO:0019843, GO:0000027, GO:0006412	−3.87
	FP0894	Translation initiation factor IF3	*infC*, FPSM_01478	GO:0003743, GO:0006413	−3.22
	FP0975	Elongation factor P (EF-P)	FPSM_01375, *efp*	GO:0005737, GO:0003746, GO:0006414	−3.20
	FP0984	Probable transcriptional regulator, AraC family	FPSM_01384, FP0984	GO:0016021, GO:0003700, GO:0043565, GO:0006351, GO:0006355	4.06
	FP0994	Probable transcriptional regulator, AraC family	FPSM_01394, FP0994	GO:0003700, GO:0043565, GO:0006351, GO:0006355	3.38
	FP1135	50S ribosomal protein L31	IA01_05395, *rpmE*	GO:0005840, GO:0003735, GO:0006412	−3.69
	FP1175	DNA-directed RNA polymerase beta' subunit RpoC	*rpoC*	GO:0003677, GO:0003899, GO:0006351	−2.65
	FP1176	DNA-directed RNA polymerase beta subunit RpoB	*rpoB*	GO:0003677, GO:0003899, GO:0032549, GO:0006351	−2.65
	FP1177	50S ribosomal protein L7/L12	*rplL*	GO:0005840, GO:0003735, GO:0006412	−3.53
	FP1178	50S ribosomal protein L10	*rplJ*	GO:0005840, GO:0070180, GO:0006412, GO:0042254	−3.98
	FP1179	50S ribosomal protein L1	*rplA*	GO:0015934, GO:0000049, GO:0003735, GO:0019843, GO:0006412, GO:0006417	−3.64
	FP1181	Transcription antitermination protein	*nusG*	GO:0006353, GO:0006354, GO:0031564, GO:0032784	−2.57
	FP1184	Elongation factor Tu (EF-Tu)	*tuf*	GO:0005737, GO:0003746, GO:0003924, GO:0005525, GO:0006414	−4.14
	FP1312	50S ribosomal protein L17	*rplQ*	GO:0005840, GO:0003735, GO:0006412	−3.40
	FP1313	DNA-directed RNA polymerase subunit alpha	*rpoA*	GO:0003677, GO:0003899, GO:0046983, GO:0006351	−4.11
	FP1314	30S ribosomal protein S4	*rpsD*	GO:0015935, GO:0003735, GO:0019843, GO:0006412	−4.55
	FP1315	30S ribosomal protein S11	*rpsK*	GO:0005840, GO:0003735, GO:0019843, GO:0006412	−4.00
	FP1316	30S ribosomal protein S13	*rpsM*	GO:0005840, GO:0000049, GO:0003735, GO:0019843, GO:0006412	−3.67
	FP1318	Translation initiation factor IF1	*infA*	GO:0005737, GO:0003743, GO:0019843, GO:0043022, GO:0006413	−3.53
	FP1320	50S ribosomal protein L15	*rplO*	GO:0015934, GO:0003735, GO:0019843, GO:0006412	−3.20
	FP1321	50S ribosomal protein L30	*rpmD*	GO:0015934, GO:0003735, GO:0006412	−4.92
	FP1322	30S ribosomal protein S5	*rpsE*	GO:0015935, GO:0003735, GO:0019843, GO:0006412	−2.91
	FP1323	50S ribosomal protein L18	*rplR*	GO:0005840, GO:0003735, GO:0019843, GO:0006412	−3.56
	FP1324	50S ribosomal protein L6	*rplF*	GO:0005840, GO:0003735, GO:0019843, GO:0006412	−4.01
	FP1325	30S ribosomal protein S8	*rpsH*	GO:0005840, GO:0003735, GO:0019843, GO:0006412	−3.60
	FP1326	30S ribosomal protein S14	*rpsN*	GO:0005840, GO:0003735, GO:0019843, GO:0006412	−3.72
	FP1327	50S ribosomal protein L5	*rplE*	GO:0005840, GO:0000049, GO:0003735, GO:0019843, GO:0006412	−4.56
	FP1328	50S ribosomal protein L24	*rplX*	GO:0005840, GO:0003735, GO:0019843, GO:0006412	−3.32
	FP1329	50S ribosomal protein L14	*rplN*	GO:0015934, GO:0003735, GO:0019843, GO:0006412	−3.11
	FP1330	30S ribosomal protein S17	*rpsQ*	GO:0005840, GO:0003735, GO:0019843, GO:0006412	−4.81
	FP1331	50S ribosomal protein L29	*rpmC*	GO:0005840, GO:0003735, GO:0006412	−5.06
	FP1332	50S ribosomal protein L16	*rplP*	GO:0005840, GO:0000049, GO:0003735, GO:0019843, GO:0006412	−3.34
	FP1333	30S ribosomal protein S3	*rpsC*	GO:0015935, GO:0003729, GO:0003735, GO:0019843, GO:0006412	−3.91
	FP1334	50S ribosomal protein L22	*rplV*	GO:0015934, GO:0003735, GO:0019843, GO:0006412	−3.47
	FP1335	30S ribosomal protein S19	*rpsS*	GO:0015935, GO:0003735, GO:0019843, GO:0006412	−3.80
	FP1336	50S ribosomal protein L2, partial	*rplB*	GO:0015934, GO:0003735, GO:0016740, GO:0019843, GO:0006412	−3.72
	FP1338	50S ribosomal protein L4	*rplD*	GO:0005840, GO:0003735, GO:0019843, GO:0006412	−3.12
	FP1339	50S ribosomal protein L3	*rplC*	GO:0005840, GO:0003735, GO:0019843, GO:0006412	−2.83
	FP1340	30S ribosomal protein S10	*rpsJ*	GO:0005840, GO:0000049, GO:0003735, GO:0006412	−2.97
	FP1341	Elongation factor G (EF-G)	*fusA*	GO:0005737, GO:0003746, GO:0003924, GO:0005525, GO:0006414	−3.89
	FP1342	30S ribosomal protein S7	*rpsG*	GO:0015935, GO:0000049, GO:0003735, GO:0019843, GO:0006412	−4.05
	FP1343	30S ribosomal protein S12	*rpsL*	GO:0015935, GO:0000049, GO:0003735, GO:0019843, GO:0006412	−3.68
	FP1397	30S ribosomal protein S20	*rpsT*	GO:0005840, GO:0003735, GO:0019843, GO:0006412	−3.51
	FP1432	50S ribosomal protein L33	*rpmG*	GO:0005840, GO:0003735, GO:0006412	−3.52
	FP1466	Extracytoplasmic function (ECF)-type sigma factor	FP1466, FPSM_01934	GO:0003677, GO:0003700, GO:0016987, GO:0006352, GO:0006355	2.79
	FP1595	Probable transcriptional regulator, ArsR family	FP1595, FPSM_01218	GO:0003677, GO:0003700, GO:0006351, GO:0006355	2.82
	FP1793	30S ribosomal protein S1	IA01_08730, *rpsA*	GO:0005840, GO:0003723, GO:0003735, GO:0006412	−3.97
	FP1794	Ribonuclease Z	FP1794		−2.06
	FP1849	50S ribosomal protein L9	*rplI*	GO:0005840, GO:0003735, GO:0019843, GO:0006412	−3.37
	FP1850	30S ribosomal protein S18	*rpsR*	GO:0005840, GO:0003735, GO:0019843, GO:0006412	−3.22
	FP1851	30S ribosomal protein S6	*rpsF*	GO:0005840, GO:0003735, GO:0019843, GO:0006412	−4.21
	FP1867	rRNA methyltransferase	FP1867, FPSM_00933	GO:0003723, GO:0008173, GO:0001510, GO:0006396	4.12
	FP2045	30S ribosomal protein S15	*rpsO*	GO:0005840, GO:0003735, GO:0019843, GO:0006412	−3.60
	FP2048	RNA polymerase sigma factor RpoD	*rpoD*, FPSM_00742	GO:0003677, GO:0003700, GO:0016987, GO:0006352, GO:0006355, GO:0030435	−3.02
	FP2052	Glutamyl-tRNA amidotransferase	FPSM_00739, FP2052	GO:0016740, GO:0016884, GO:0008152	−2.16
	FP2124	Epoxyqueuosine reductase (tRNA modification)	*queG*, FP2124	GO:0005737, GO:0046872, GO:0051539, GO:0052693, GO:0008033, GO:0008616, GO:0055114	3.97
	FP2216	50S ribosomal protein L27	*rpmA*	GO:0005840, GO:0003735, GO:0006412	−3.29
	FP2217	50S ribosomal protein L21	*rplU*	GO:0005840, GO:0003677, GO:0003735, GO:0019843, GO:0006281, GO:0006412	−3.41
	FP2380	50S ribosomal protein L32	*rpmF*	GO:0015934, GO:0003735, GO:0006412	−2.38
DNA repair and antioxidant activity	FP0160	Superoxide dismutase	FPSM_00176, *sodA*	GO:0004784, GO:0046872, GO:0019430, GO:0055114	−2.38
	FP0209	Crossover junction endodeoxyribonuclease	*ruvC*	GO:0000287, GO:0003676, GO:0008821, GO:0006281, GO:0006310, GO:0090305	2.61
	FP0269	DNA repair protein RecO	*recO*	GO:0006281, GO:0006310	3.78
	FP0568	Cytochrome C peroxidase	FP0568	GO:0004130, GO:0009055, GO:0020037, GO:0055114	5.37
	FP0670	Chaperone protein DnaJ	*dnaJ*	GO:0005737, GO:0005524, GO:0008270, GO:0031072, GO:0051082, GO:0006260, GO:0006457, GO:0009408	−3.16
	FP0671	Chaperone protein GrpE	*grpE*	GO:0005737, GO:0000774, GO:0042803, GO:0051087, GO:0006457, GO:0050790	−2.87
	FP0702	Probable peroxiredoxin	FPSM_01619, FP0702	GO:0004601, GO:0051920, GO:0055114	2.37
	FP0864	Chaperone protein DnaK	*dnaK*	GO:0005524, GO:0051082, GO:0006457	−4.00
	FP1014	Deoxyribodipyrimidine photolyase PhrB1	*phrB1*, IA01_04785	GO:0003904, GO:0018298	3.99
	FP1100	Thiol peroxidase	FPSM_02014, FP1100	GO:0004601, GO:0051920, GO:0055114	−2.05
	FP1195	DNA polymerase III subunit epsilon	*dnaQ*, FPSM_02105	GO:0003676, GO:0003887, GO:0004527, GO:0071897, GO:0090305	3.53
	FP1417	Glutathione peroxidase	FPSM_01888, *bsaA*	GO:0004602, GO:0006979, GO:0055114	−2.63
	FP1509	Chaperone protein HtpG	*htpG*, IA01_07270	GO:0005737, GO:0005524, GO:0051082, GO:0006457, GO:0006950	−3.32
	FP1594	2-Cys peroxiredoxin	*tpx*	GO:0005623, GO:0008379, GO:0045454, GO:0055114	−2.01
	FP1984	groS chaperonin GroES	*groS*	GO:0005737, GO:0005524, GO:0006457	−3.48
	FP1985	groL chaperonin GroEL	*groL*, groEL	GO:0005737, GO:0005524, GO:0051082, GO:0042026	−3.03
	FP2103	Probable thioredoxin	*yyaL*, FPSM_00685	GO:0004798, GO:0046939	2.82
	FP2335	Thioredoxin family protein precursor	FPSM_02461	GO:0003756, GO:0006457	−3.72
	FP2336	Thioredoxin family protein	FP2336	GO:0005623, GO:0045454	−2.76
	FP2437	DNA processing Smf protein	FPSM_02567, *smf*	GO:0003677, GO:0006281, GO:0009294	3.77
Electron transport	FP0335	Electron transfer flavoprotein, alpha subunit	FPSM_00371, *etfA*	GO:0009055, GO:0050660	−2.80
	FP0336	Electron transfer flavoprotein, beta subunit	FP0336	GO:0009055	−3.16
	FP0376	Alternative complex III, protein E precursor	FPSM_00413, *actE*	GO:0009055, GO:0020037	−3.44
	FP0377	Alternative complex III, protein F	*actF*, FPSM_00414	GO:0016021	−2.12
	FP0378	Cytochrome C oxidase, subunit II	FPSM_00415, *ctaC*	GO:0016021, GO:0070469, GO:0004129, GO:0005507, GO:0022900, GO:1902600	−2.76
	FP0379	Cytochrome C oxidase	*ctaD*, FPSM_00416	GO:0016021, GO:0070469, GO:0004129, GO:0005506, GO:0020037, GO:0009060, GO:1902600	−2.64
	FP1428	Putative thiol: disulfide oxidoreductase TlpA	IA01_06860, *tlpA*	GO:0016491, GO:0055114	−3.13
	FP2108	Cytochrome oxidase subunit III	FP2108, FPSM_00680	GO:0016021, GO:0004129, GO:0019646, GO:1902600	−3.34
ATP synthesis	FP0114	ATP synthase subunit beta	*atpD*	GO:0005886, GO:0045261, GO:0005524, GO:0046933, GO:0015991, GO:0042777	−3.05
	FP0115	ATP synthase subunit epsilon	FPSM_00124, *atpC*	GO:0045261, GO:0046933, GO:0046961, GO:0015986	−3.05
	FP2457	ATP F0F1 synthase subunit gamma	*atpG*	GO:0005886, GO:0045261, GO:0005524, GO:0046933, GO:0046961, GO:0042777	−2.34
	FP2458	ATP F0F1 synthase subunit alpha	*atpA*	GO:0005886, GO:0045261, GO:0005524, GO:0046933, GO:0046961, GO:0015991, GO:0042777	−3.05
	FP2459	ATP synthase subunit delta	*atpH*	GO:0005886, GO:0045261, GO:0046933, GO:0042777	−2.06
	FP2460	ATP F0F1 synthase subunit B	*atpF*	GO:0005886, GO:0016021, GO:0045263, GO:0046933, GO:0042777	−4.45
	FP2461	ATP synthase subunit C	*atpE*, FPSM_02597	GO:0005886, GO:0016021, GO:0045263, GO:0015078, GO:0016787, GO:0015986, GO:0015991	−3.22

*Fold-change values (Log_2_ FC) for any differentially expressed candidate gene (DECG) in the mature biofilm state are written with positive and negative numbers to represent up- and down-regulation, respectively*.

*Up- and down-regulated candidate genes in biofilm cells are down- and up-regulated in planktonic cells, respectively*.

(*) *Locus tags according to the reference genome (F. psychrophilum strain JIP02/86) used for high-quality read mapping*.

(**) *Hit sequences collected using the BLAST algorithm run against the non-redundant database at NCBI*.

(***) *Padj-values ≤ 0.001*.

### Potential adaptive response to stress of *F. psychrophilum* biofilms

Most DECGs linked to protein synthesis were significantly down-regulated in mature biofilms of *F. psychrophilum* LM-02-Fp and NCMB1947^T^. Specifically, those encoding ribosomal proteins (*n* = 47), initiation factors IF1 and IF3 (FP1318 and FP0894, respectively), and elongation factors EF-Ts, EF-P, EF-Tu, and EF-G (FP0453, FP0975, FP1184, and FP1341, respectively) (Table 1). The same pattern was found for some DECGs encoding proteins involved in cell survival induced by growth arresting (universal stress protein UspA, FP0467); the prevention of functional ribosome formation (FP0602); the maturation of tRNAs (FP1794); and RNA synthesis using DNA as a template (FP1175, FP1176, and FP1313) (Table 1). Furthermore, transcriptional factors DksA (RNA polymerase-binding transcription factor, FP0218) and NusG (transcriptional elongation factor, FP1181), as well as the sigma factor RpoD (primary sigma factor during exponential growth of bacteria, FP2048), were significantly down-regulated in mature biofilms (Table 1). In contrast, DECGs encoding transcriptional factors from the families AraC (FP0253, FP0984, and FP0994), TetR (FP0423), LysR (FP0763), and ArsR (FP1595) were significantly up-regulated (Table 1). The same was true for DECGs encoding the extracytoplasmic function-type sigma factor (FP1466), an rRNA methyltransferase (FP1867), and the epoxyqueuosine reductase (FP2124).

Four DECGs encoding proteins required for DNA repair (FP0209/RuvC, FP0269/RecO, FP1014/PhrB1, and FP2437/Smf) and the editing exonuclease subunit of DNA polymerase III (epsilon, FP1195/DnaQ) were significantly up-regulated in mature biofilms of the two *F. psychrophilum* strains (Table 1). Similarly, DECGs encoding cytochrome C peroxidase (FP0568), a probable peroxiredoxin (FP0702), and a probable thioredoxin (FP2103) were significantly up-regulated in biofilm cells, although other antioxidant-coding DECGs (FP0160, FP1100, FP1417, FP1594, FP2335, and FP2336) were under-expressed (Table 1). DECGs associated with stress-induced responses due to heat and/or osmotic shocks (FP0670, FP0671, FP0864, FP1509, FP1984, and FP1985) were also down-regulated in mature biofilms (Table 1). Finally, eight DECGs encoding electron transport proteins with roles in the formation of transmembrane electrochemical potential were down-regulated in biofilm cells, just as were seven DECGs with roles in ATP synthesis (as part of the ATP synthase complex) (Table 1).

### Evaluation of the virulence of sessile and planktonic cells of *F. psychrophilum*

**Figure 3:**
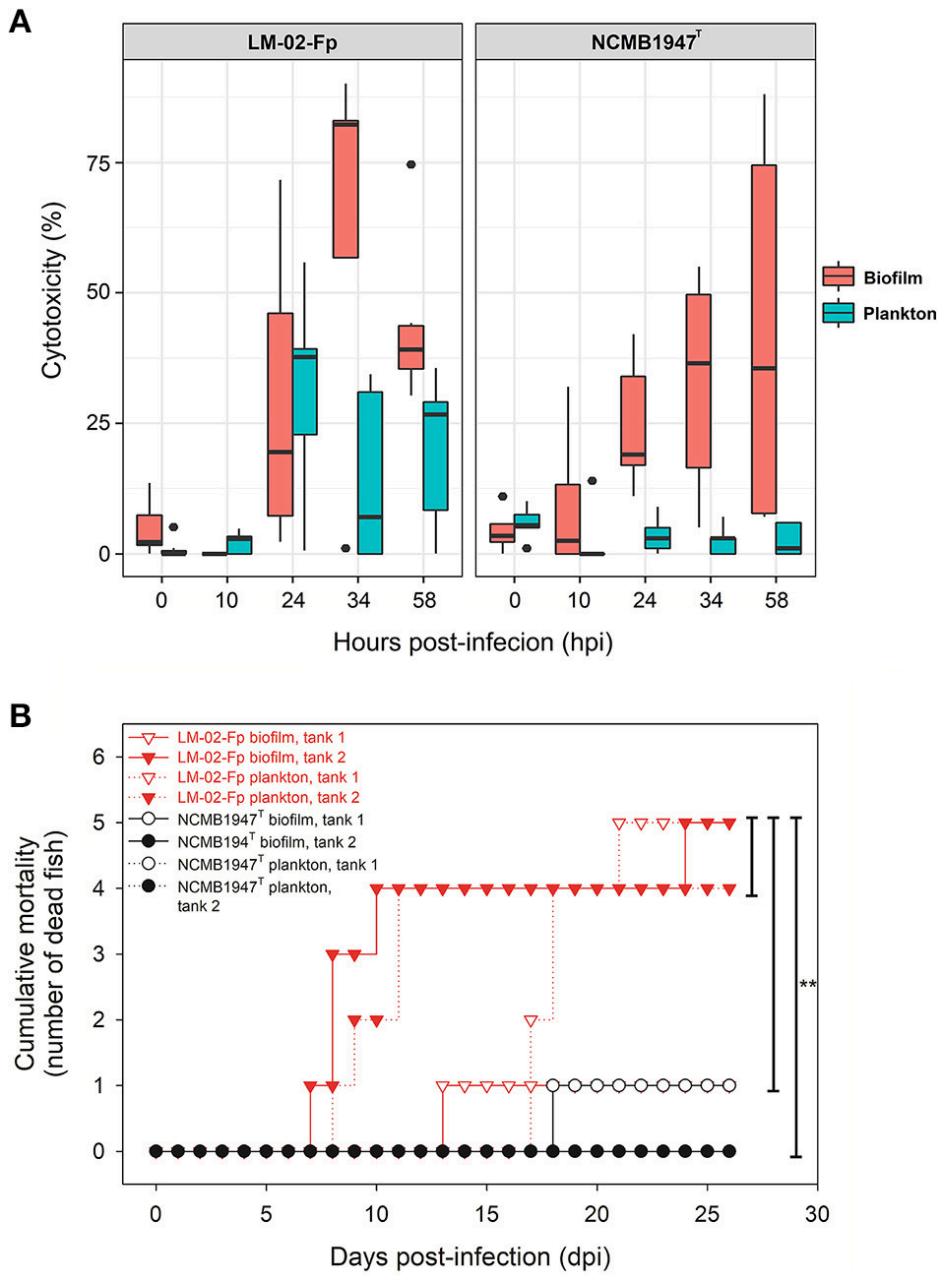
Evaluation of virulence properties of planktonic and biofilm cells of *F. psychrophilum* LM-02-Fp and NCMB1947^T^. **(A)** Box plot shows the *F. psychrophilum*-induced cytotoxicity on CHSE-214 cells in three independent experiments performed in triplicate for every strain and growth condition. The median of data set, upper/lower quartiles (boxes), range of percentages (vertical lines), and extreme values (black circles) are shown. **(B)** Cumulative mortality in rainbow trout fry after a 26-days intraperitoneal challenge with sessile and planktonic cells. No dead fish were registered in control tanks (data not shown). Five fish inoculated with LM-02-Fp biofilm cells (tank 1) died by suffocation within the first 2 h after inoculation because of malfunctioning aeration pump; results for this experimental tank are shown on figure but not considered in statistical analyses. Statistically significant differences (*P* < 0.05) are indicated by asterisks.

LDH-based assays showed that *F. psychrophilum* biofilms had higher cytotoxic effects than free-living bacteria in general (Figure 3A). The cytotoxic effect of bacteria on the fish cell line tended to increase with the time post-infection, except for infections performed with NCMB1947^T^ planktonic cells (Figure 3A). Statistical models computed by GAMLSS indicated that ~18% of the variability in the cytotoxicity caused by *F. psychrophilum* NCMB1947^T^ and LM-02-Fp was significantly explained by the growth state (biofilm vs. plankton) and time post-infection, but not by the bacterial strain (model 1, Supplementary Table 4). The time post-infection was the most significant predictor variable for the strain LM-02-Fp (model 2, Supplementary Table 4), although an important increase in the cytotoxicity induced by LM-02-Fp biofilm cells was detectable after 24 hpi compared with planktonic cells (Figure 3A). In addition, the growth state as well as its interaction with time post-infection, were the most significant predictor variables for the strain NCMB1947^T^ (model 3, Supplementary Table 4).

There were no significant intrastrain differences in generating rainbow trout fry mortality between planktonic and sessile cells of *F. psychrophilum* (Figure 3B). The same was true for differences in fish mortality found between NCMB1947^T^ and LM-02-Fp strains, except when the type strain did not produce mortalities (*P* < 0.05, Gehan-Breslow-Wilcoxon and Log-rank tests), situation observed in biofilm tank 1 and plankton tank 2 (Figure 3B). No fish mortality was observed during experiments in control tanks and microbiological/PCR analyses confirmed *F. psychrophilum*-caused mortality in challenged fish (data not shown).

## Discussion

Transcriptomes of *F. psychrophilum* LM-02-Fp and NCMB1947^T^ grown under biofilm and planktonic conditions were made up of high-quality reads for a total of 2327 genes, 1918 of which (i.e., ~82% of total number of genes expressed in transcriptomes) did not show statistically significant differences in gene expression between growth states (Levipan and Avendaño-Herrera, 2017). The present study reports 349 DECGs (Figure 1) accounting for ~15% of total number of genes expressed in transcriptomes, since other 60 DECGs have been described previously as differentially expressed virulence- and biofilm-related genes (Levipan and Avendaño-Herrera, 2017). A similar number of differentially expressed genes (*n* = 440) was determined through DNA arrays in *Escherichia coli* K-12 TG1 by comparing 8-day-old biofilms with planktonic cells in the late exponential stage (Beloin et al., 2004). In our study, DECGs encoding ribosomal proteins, some transcriptional factors, different subunits of the DNA-directed RNA polymerase and ATP synthase, as well as translation and elongation factors were significantly down-regulated in mature biofilms as compared to free-living cells (Figure 1 and Table 1). These findings might have important implications in selecting the candidate antigens for vaccine design, since certain antigenic phenotypes could change over time through the cell cycle, depending on whether bacteria are growing in planktonic or biofilm modes. Thus, a vaccine designed from planktonic cells (killed and/or live-attenuated; e.g., Álvarez et al., 2008) or purified macromolecules may be ineffective to deal with biofilm-related infections, where the planktonic phenotype is only a transient state (Harro et al., 2010). For instance, elongation factor-Tu and ATP synthase subunit beta, previously reported as immunogenic proteins (LaFrentz, 2007), were unsuitable as vaccine against BCWD/RTFS (Plant et al., 2011), which could be related with the fact that these proteins are coded by DECGs between biofilm and planktonic states in *F. psychrophilum* (Table 1). Indeed, the antigenic variability between biofilm and planktonic states represents a so far unexplored mechanism (Gómez et al., 2014) that could explain the persistence of this bacterium in aquaculture settings.

Some regulator-coding genes of cellular persistence-related genes (e.g., AraC family transcriptional regulators and extracytoplasmic function-type sigma factor) with putative functions in carbon metabolism, virulence, and diverse stress responses, such as metal-ion tolerance (ArsR-like transcriptional factor), were significantly up-regulated in mature biofilms (Table 1). Indeed, biofilm lifestyle is well known for providing bacteria with improved protection against varied physical-chemical stressors, that is, nutrient depletion, UV radiation, reactive oxygen species, and antibiotics (Flemming and Wingender, 2010). For instance, the overexpression of a rRNA methyltransferase in *F. psychrophilum* biofilms (FP1867, Table 1) could confer greater resistance to antibiotics targeting the ribosomes (Doi and Arakawa, 2007) to sessile cells than free-living cells. Moreover, the improved protection of bacteria in biofilm growth mode is usually accompanied by enhanced virulence and pathogenicity (He and Ahn, 2011; Flemming et al., 2016). However, there are few studies on *F. psychrophilum* comparing free-living vs. biofilm lifestyles (e.g., De la Fuente et al., 2013), and research based on mutants with defects in motility (Álvarez et al., 2006; Pérez-Pascual et al., 2015) has reported an attenuation of virulence in spread-deficient mutants compared to wild-type strains (Pérez-Pascual et al., 2017). These findings suggest that *F. psychrophilum* biofilm cells could be less virulent than motile free-living cells; however, this suggestion overlooks the fact that *F. psychrophilum* infections rely on initial attachment to mucosal external surfaces of fish (Papadopoulou et al., 2017) for cell colonization and long-term persistence of adhered cells. We have previously reported that strong and weak biofilm producers (LM-02-Fp and NCMB1947^T^ strains in the present study, respectively) share a genetic potential for virulence that is transcriptionally enhanced with respect to free-living cells (Levipan and Avendaño-Herrera, 2017). Here we have found that biofilm and planktonic states of *F. psychrophilum* have strain-independent cytotoxic effects on CHSE-214 cells (Figure 3A), with this being particularly true for the sessile lifestyle (Supplementary Figure 1 and Supplementary Table 4). Chinook embryonic cells have successfully been used for studying adhesion to host of extracellular fish pathogens such as *Streptococcus phocae* (González-Contreras et al., 2011), and there is at least one previous study describing *F. psychrophilum*-induced degenerative changes on CHSE-214 cells (Valdebenito and Avendaño-Herrera, 2009). Moreover, even though our intraperitoneal challenge model did not support the idea that differences between sessile and planktonic lifestyles are associated with state-dependent intrastrain differences in virulence, the strong biofilm producer tended to produce higher mortality in rainbow trout fry than the type strain (Figure 3B). This trend and LDH-based cytotoxicity were associated with lower transcriptional/translational activities for growth in biofilm cells than in free-living cells (Figure 1) at the moment of initial infection. This was also reflected as a lower percentage contribution of up-regulated candidate genes in the biofilm state to the top-level GO term “cellular component,” as compared to down-regulated candidate genes (Figure 2). However, it is also important to note that the growth of 96-h-old biofilms was reduced but not arrested, since an increased percentage contribution of up-regulated candidate genes was observed for “molecular function” and “biological processes” ontologies (Figure 2). For instance, the *uspA* gene encoding universal stress protein (FP0467), that is usually over-expressed under growth arrest for cell survival (Nachin et al., 2005), was significantly down-regulated in mature biofilms of *F. psychrophilum* LM-02-Fp and NCMB1947^T^ (Figure 1).

Mature *F. psychrophilum* biofilms showed a significant up-regulation of all DECGs with roles in DNA repair, namely, *ruvC, recO, smf*, *phrB1*, and *dnaQ* (Table 1). Overexpression of genes and proteins involved in DNA repair has also been reported for *F*. *johnsoniae* biofilms (Flemming, 2010), suggesting shared strategies between fish pathogenic bacteria in the biofilm state to face environmental changes. The *ruvC* and *recO* genes are involved in RecA-dependent homologous recombinational repair (via the RecF pathway or RecFOR) to handle single-stranded gaps in DNA (Persky and Lovett, 2008). In turn, the *smf* gene product is important for extracellular DNA uptake and protection during recombinational repair (Smeets et al., 2006). DNA transformation and recombinational repair are processes consistent with the biofilm lifestyle of *F. psychrophilum*, since extracellular DNA is a major structural component in most bacterial biofilms and substrates for lateral gene transfer events (Flemming and Wingender, 2010; Levipan and Avendaño-Herrera, 2017). The *phrB1* gene encodes a photolyase that corrects UV radiation-induced cyclobutane pyrimidine dimers in the *F. psychrophilum* genome (Goosen and Moolenaar, 2008), while *dnaQ* encodes the epsilon-subunit of DNA polymerase III, which mediates 3′-5′ proofreading in this enzyme. Up-regulation of *dnaQ* decreases the bacterial mutation rate during the SOS response (e.g., Jonczyk et al., 1988). Thus, considering that SOS response plays a key role in biofilm formation and maturation (van der Veen and Abee, 2010; Leiker and Weitao, 2016), overexpression of the *dnaQ* gene would be expected in mature *F. psychrophilum* biofilms. In practice, these findings could be associated with the relative ineffectiveness of UV radiation-based technologies for *F. psychrophilum* removal from and the prevalence of BCWD/RTFS in aquaculture settings, since a 5-log reduction in cell abundance of this bacterium can need UV doses as high as 126 mW s cm^−2^ (Hedrick et al., 2000).

Among nine of the DECGs encoding for molecular markers of oxidative stress (Table 1), only cytochrome C peroxidase (FP0568), a probable peroxiredoxin (FP0702), and a probable thioredoxin (FP2103) were significantly up-regulated in mature biofilms to deal with reactive oxygen species (ROS, e.g., H_2_O_2_). This finding suggests two possibilities, (i) that the degree of oxidative damage during biofilm formation was insufficient to induce a joint overexpression of these 9 DECGs as a single and coordinated scavenging system of ROS, or (ii) that a different set of proteins copes with oxidative stress in biofilm cells, as compared to planktonic cells. Nevertheless, oxidative damage should have a bacteriostatic rather than bactericidal effect in bacterial cells whether stress-defense mechanisms are fully functional (e.g., DNA repair; Imlay, 2015). Recently, it has been hypothesized that biofilms exploit ROS to activate signaling pathways that affect the production of extracellular polymeric substances and biofilm heterogeneity to adapt to changing conditions (Gambino and Cappitelli, 2016). For instance, sub-inhibitory concentrations of Ag-NPs stimulated the production of ROS in *Bacillus subtilis* biofilms but not in planktonic cells, which was also accompanied by the expression of proteins involved in quorum sensing and oxidative stress response in sessile cells, including thioredoxin (Gambino et al., 2015). Therefore, if thioredoxin-like proteins (in our case FP2103) are involved (via ROS) in signaling that controls the biofilm formation by *F. psychrophilum*, for example, through two-component systems (Hesami et al., 2011; Levipan and Avendaño-Herrera, 2017), is a topic that remains to be elucidated. On the other hand, DECGs encoding proteins with roles in the adaptive response of *F. psychrophilum* to temperature shifts (Duchaud et al., 2007) such as chaperone proteins DnaJ (FP0670), GrpE (FP0671), DnaK (FP0864), HtpG (FP1509), and GroS/GroL chaperonin GroES/GroEL (FP1984 and FP1985, respectively) were significantly down-regulated in mature biofilms (Table 1). These stress-induced proteins ensure cell protein homeostasis by preventing polypeptide aggregation and misfolding (Hartl et al., 2011). Therefore, under a condition of overall reduction of protein synthesis machinery in *F. psychrophilum* biofilms (Table 1), a significant expression of DECGs encoding products with roles in polypeptide homeostasis would mean an unnecessary energetic cost for sessile cells, which could explain these findings.

DECGs encoding respiratory complex-related proteins and different subunits of the ATP synthase were significantly down-regulated in mature biofilms (Table 1), suggesting a negative relationship between oxidative phosphorylation for energy conservation and maintenance of biofilm structure. In agreement with this finding, a study based on *ycfR*-deletion mutants of *E*. *coli* K-12 reported a transcriptional repression of several genes encoding ATP synthase subunits that coincided with an enhanced capacity to form biofilms (Zhang et al., 2007). This work along with our study suggest that a decreased capacity for ATP energy conservation does not necessarily leads to the early and total disaggregation of bacterial biofilms, as could be deduced from other bacterial models (Sule et al., 2009) or experiments with multi-species biofilms where the ATP synthesis was chemically inhibited (Xu et al., 2012). In fact, the earliest cell detachment in our experiments began at approximately 144 h of incubation post-inoculation, and biofilm cells at 268 h of incubation were still detectable (Levipan and Avendaño-Herrera, 2017). This latter suggests that a down-regulation of DECGs related to energy conservation represents a blocking cue for energy-demanding processes (e.g. protein synthesis) rather than triggering cell detachment from mature *F. psychrophilum* biofilms.

In summary, this study unveiled the major patterns of shift in overall gene expression induced by plankton-to-biofilm transition in *F. psychrophilum*. This report represents the first transcriptomic baseline toward obtaining insights into the biofilm lifestyle of this freshwater fish pathogen. Specifically, a potential stress response in mature biofilms was identified, as characterized by a generalized under-expression of DECGs with roles in protein synthesis machinery and ATP-based energy conservation via oxidative phosphorylation, along with an overexpression of several DECGs with roles in preserving cell integrity. This pattern suggests that mature *F. psychrophilum* biofilms employ an energetically economical strategy to ensure structural maintenance and metabolic functioning, relying on practically no growth and the induction of cellular homeostasis processes such as DNA repair. Finally, our results propose new avenues of research that may help to understand and/or face the prevalence of *F. psychrophilum* in aquaculture settings: (1) pathogenicity and virulence of the biofilm lifestyle, (2) changes between planktonic and biofilm states that lead to intra-strain antigenic variations with potential impact on vaccine design, (3) role of oxidative stress-related proteins in biofilm formation, and (4) energy conservation and its implication for biofilm deterioration.

## Ethics statement

This study adhered to animal welfare procedures and was approved by the bioethical committees of the Universidad Andrés Bello and the National Commission for Scientific and Technological Research of the Chilean government.

## Author contributions

HL and RA-H conceived and designed the study. JQ performed cytotoxicity experiments. HL conducted the rest of experiments, performed the analysis and interpretation of data, and wrote the manuscript. RA-H participated in the critical review of the manuscript.

### Conflict of interest statement

The authors declare that the research was conducted in the absence of any commercial or financial relationships that could be construed as a potential conflict of interest.
