# Supplementary material for: Stress Tolerance-Related Genetic Traits of Fish Pathogen Flavobacterium psychrophilum in a Mature Biofilm
Source: Front Microbiol. 2018 Jan 23;9:18. doi: 10.3389/fmicb.2018.00018 (PMC5787105; doi:10.3389/fmicb.2018.00018)
Supplement: Supplementary file 1 [file Table1.pdf]

Supplementary Material

Stress Tolerance-Related Genetic Traits of Fish Pathogen *Flavobacterium psychrophilum* in a Mature Biofilm

Héctor A. Levipan, Johan Quezada, and Ruben Avendaño-Herrera

1.1 Supplementary Table 1

Table S1. Primers used for amplifying genes potentially associated with stress response in *F. psychrophilum*. Names based on locus tags according to the *F. psychrophilum* JIP02/86 genome, description of target genes, amplicon length, primer sequences, and annealing temperatures.

Primer name	Gene description (gene symbol)	Amplicon (bp)	Sequence (5' to 3')	Annealing temperature (ºC)
FP0269-F	DNA repair protein RecO (*recO*)	156	CGCTTTGCATTGGTTAGACA	66
FP0269-R			GCGTGAAAGGGAGAAAACAG	
FP0335-F	Electron transfer flavoprotein, alpha subunit (*etfA*)	188	CCCAAATAAATACCGACATAA	62
FP0335-R			GACTACTACATCAGCATCT	

FP0453-F	Elongation factor Ts, EF-Ts (*tsf*)	155	GTAGCTGCAAACCGTTCAGA	66
FP0453-R			GCCATTTCAACAGCCATTTT	
FP0467-F	Universal stress protein UspA (*uspA*)	184	CGAGCACGCCCTTAAAGTAG	66
FP0467-R			ATCTTGCGCCATCAAATCTT	
FP0671-F	Chaperone protein GrpE (*grpE*)	194	TACAAGCCATGTTGCCAGTT	62
FP0671-R			AATTGCTTCGGCAAAATCTG	
FP0892-F	50S ribosomal protein L20 (*rplT*)	151	AATGAAGCAAGCCAAAGGTT	66
FP0892-R			AGCGTTGATACGCTGAATCC	
FP1175-F	DNA-directed RNA polymerase beta' subunit RpoC (*rpoC*)	195	CCGATGCAAGAGCACAAGTA	67
FP1175-R			TTGCACCAGAGTCAAGCATC	
FP1181-F	Transcription antitermination protein (*nusG*)	209	GCGGAAATTAACAGGCTAGAA	66
FP1181-R			TTTGTTTCGCCAAGAAAACC	
FP2458-F	ATP F0F1 synthase subunit alpha (*atpA*)	186	ACCRGGACGTGARGCATATC	66
FP2458-R			CCCGCTTGTGTTTCGATAAT	

1.2 Supplementary Table 2

Table S2. Sample information and summary of transcriptome sequencing data for *F. psychrophilum* strains under planktonic and biofilm states.

	**Planktonic**		**Biofilm**	
	NCMB1947^T	LM-02-Fp	NCMB1947^T	LM-02-Fp
RIN score	9.3	9.2	6.3	7.1
ENA sample	ERS1231641	ERS1231642	ERS1231643	ERS1231644
Read number	11126353	10552442	5574009	6777655
Average read length (bp)	79.6	79.5	121.5	78.8
Read number after quality filtering	10246685	9559727	4875335	5956521
Average read length after quality filtering (bp)	67.7	67.4	110.3	67.1
Mapped reads	8215988	6524164	60666	152711

1.3 Supplementary Table 3

Table S3. DECGs between biofilm and planktonic states of *F. psychrophilum* LM-02-Fp and NCMB1947^{T}. Locus tags were assigned to each DECG according to the annotations for the *F. psychrophilum* JIP02/86 reference genome. Only statistically significant fold-change values are shown (Padj ≤ 0.001).

Locus Tag	DECG description	Hit name*	GO term number	Log_2 fold-change
FP0049	Probable 3-hydroxybutyryl-CoA dehydratase	FPSM_00051, *crt*, FP0049	GO:0003859, GO:0008152	-2.05
FP0051	Putative acyl-[acyl-carrier-protein] desaturase	FPSM_00053, FP0051	GO:0045300, GO:0006631, GO:0055114	-2.23
FP0093	Succinate dehydrogenase, flavoprotein subunit	*sdhA*	GO:0008177, GO:0055114	-2.32
FP0095	Fumarate reductase	IA01_00470, *sdhB*	GO:0000104, GO:0009055, GO:0051537, GO:0055114	-2.18
FP0111	Glutamine aminotransferase	*glmS*	GO:0005737, GO:0004360, GO:0030246, GO:0005975, GO:0006541, GO:1901137	-2.34
FP0123	Phenylalanyl-tRNA synthetase subunit alpha	IA01_00605	GO:0004812, GO:0008152	2.12
FP0136	ATPase, MoxR family	FPSM_00145, FP0136	GO:0005524, GO:0016887, GO:0008152	-2.49
FP0142	Aldehyde reductase	FPSM_00151, FP0142	GO:0016491, GO:0046872, GO:0055114	-2.77
FP0144	Probable TonB-dependent outer membrane receptor precursor	FP0144	GO:0016020, GO:0004872, GO:0006810	-2.50
FP0147	Ribose-phosphate pyrophosphokinase	*prsA*, FPSM_00156	GO:0000287, GO:0004749, GO:0016301, GO:0009156, GO:0009165, GO:0016310	-3.44
FP0153	Xaa-Pro aminopeptidase	*pepP*, FPSM_00169	GO:0004177, GO:0030145, GO:0006508	-2.87

FP0186	Nicotinamide mononucleotide transporter PnuC	SU65_00985, *pnuC*	GO:0016021, GO:0034257, GO:0034258	2.78
FP0187	Geranylgeranylglyceryl phosphate synthase (PcrB protein homolog)	*pcrB*, IA01_00895	GO:0005737, GO:0000287, GO:0003949, GO:0047294, GO:0006650	2.73
FP0201	3-oxoacyl-ACP synthase	*fabF*, FPSM_00227	GO:0033817, GO:0006633	-2.37
FP0208	Coproporphyrinogen III oxidase	FP0208, FPSM_00234	GO:0005737, GO:0004109, GO:0051536, GO:0051989, GO:0006779, GO:0055114	3.33
FP0210	Hypothetical protein	FP0210, IA01_01015	GO:0016021	4.58
FP0211	Beta-lactamase regulatory	FPSM_00237, FP0211	GO:0016021, GO:0016757, GO:0008152	5.24
FP0213	Hypothetical protein (membrane)	FPSM_00239, FP0213	GO:0016021	4.64
FP0241	3-phosphoshikimate 1-carboxyvinyltransferase	*aroA*	GO:0005737, GO:0003866, GO:0009073, GO:0009423	2.27
FP0249	Putative phospho-2-dehydro-3-deoxyheptonate aldolase	FP0249, FPSM_00276	GO:0003849, GO:0004106, GO:0009058, GO:0046417	2.13
FP0250	Probable aspartate transaminase AspC3	*aspC3*, FPSM_00277	GO:0004069, GO:0030170, GO:0080130, GO:0009058	2.77
FP0255	Aspartate kinase	FPSM_00282, FP0255	GO:0004072, GO:0004412, GO:0050661, GO:0009086, GO:0009088, GO:0009097, GO:0016310, GO:0055114	4.26
FP0256	Homoserine O-acetyltransferase	IA01_01250, *metX*, FP0256	GO:0005737, GO:0004414, GO:0009086	5.88
FP0257	Probable O-acetylhomoserine aminocarboxypropyltransferase	*metY*, FP0257	GO:0003961, GO:0030170, GO:0071266	5.75
FP0267	Protein of unknown function precursor	FP0267, IA01_01300	GO:0016020, GO:0004872, GO:0006810	3.63
FP0271	Glutamate dehydrogenase (NADP+)	*gdhA*, IA01_01320	GO:0016639, GO:0006520, GO:0055114	-3.02
FP0282	Deoxyguanosinetriphosphate triphosphohydrolase (dGTPase)	*dgt*, FPSM_00316	GO:0000287, GO:0008832, GO:0046039	2.08
FP0313	Mevalonate kinase	*mvaK*, FPSM_00349	GO:0005737, GO:0004496, GO:0005524, GO:0016310	-2.13
FP0323	Fructose-bisphosphate aldolase	*fbaA*, FPSM_00359	GO:0004332, GO:0008270, GO:0006096	-2.03
FP0347	Glutaryl-CoA dehydrogenase	*gcdH*, IA01_01740	GO:0003995, GO:0050660, GO:0055114	-2.19

FP0354	Cys/Met metabolism PLP-dependent enzyme	FPSM_00391, FP0354	GO:0004123, GO:0030170, GO:0044540, GO:0080146, GO:0008152	-3.23
FP0357	Probable TonB-dependent outer membrane receptor precursor	FPSM_00394, FP0357	GO:0016020, GO:0004872, GO:0006810	3.26
FP0367	Hypothetical protein (phosphoesterase)	FPSM_00404, FP0367	GO:0016787, GO:0008152	3.06
FP0411	Phosphoribosylglycinamide formyltransferase	*purT*	GO:0000287, GO:0004644, GO:0005524, GO:0043815, GO:0006189	2.54
FP0415	1-pyrroline-5-carboxylate dehydrogenase	FPSM_00452, *pruA*	GO:0003842, GO:0016620, GO:0006537, GO:0006561, GO:0055114	-3.01
FP0422	ABC transporter ATP-binding	FPSM_00465, FP0422	GO:0016021, GO:0005524, GO:0042626, GO:0008152, GO:0055085	2.12
FP0424	4-hydroxybutyrate coenzyme A transferase	*cat2*, FPSM_00467	GO:0016740, GO:0006084	-2.04
FP0428	Thiamine biosynthesis protein ThiC	*thiC*	GO:0008270, GO:0016829, GO:0051539, GO:0009228, GO:0009229	2.14
FP0431	Thiamine-phosphate synthase	*thiE*	GO:0000287, GO:0004789, GO:0009228, GO:0009229	3.35
FP0432	Thiazole biosynthesis protein ThiG	*thiG*	GO:0005737, GO:0016783, GO:0036355, GO:0009228, GO:0009229	3.43
FP0434	Molybdopterin and thiamine biosynthesis protein	*thiF*, FPSM_00477	GO:0008641, GO:0008152	2.84
FP0441	Probable Na+/H+ antiporter	FPSM_00484, FP0441	GO:0016021, GO:0015299, GO:1902600	2.37
FP0445	Threonine dehydratase	*ilvA*	GO:0004794, GO:0030170, GO:0009097	4.14
FP0446	ketol-acid reductoisomerase	*ilvC*, FPSM_00489	GO:0004455, GO:0016853, GO:0009097, GO:0009099, GO:0055114	5.38
FP0448	Acetolactate synthase catalytic subunit	FPSM_00491, *ilvB*	GO:0000287, GO:0003984, GO:0030976, GO:0050660, GO:0009097, GO:0009099	5.72
FP0449	Dihydroxy-acid dehydratase	FP0449	GO:0004160, GO:0046872, GO:0051539, GO:0009097, GO:0009099	6.08
FP0450	Branched-chain-amino-acid transaminase	*ilvE*, FPSM_00494	GO:0052654, GO:0052655, GO:0052656, GO:0009081	5.68
FP0507	Tryptophan synthase, alpha subunit		GO:0004834, GO:0000162	2.32

FP0512	Para-aminobenzoate/anthranilate synthase glutamine aminotransferase component II	IA01_02580, *pabA*	GO:0004049, GO:0046820, GO:0006541	2.67
FP0526	Asparagine synthetase B	FP0526, FPSM_01811	GO:0004066, GO:0006529	2.79
FP0528	Malate dehydrogenase	*mdh*	GO:0030060, GO:0005975, GO:0006099, GO:0006108	-2.35
FP0532	ABC transporter	FPSM_01805, FP0532	GO:0016021, GO:0005524, GO:0042626, GO:0008152, GO:0055085	2.48
FP0533	Valine dehydrogenase	FPSM_01804, *vdh*	GO:0050049, GO:0050391, GO:0006520, GO:0055114	-4.14
FP0565	Hypothetical protein	FPSM_01769, FP0565	GO:0005509, GO:0016462, GO:0008152	3.52
FP0581	Pre-translocase subunit	*secA*	GO:0005737, GO:0005886, GO:0005524, GO:0006605, GO:0017038, GO:0065002	-2.09
FP0591	Hypothetical protein	FP0591, FPSM_01741	GO:0016021	4.53
FP0593	Hypothetical protein FPSM_01739	FP0593	GO:0003677	2.02
FP0603	Cell division protein FtsH	*ftsH*	GO:0005886, GO:0016021, GO:0004222, GO:0005524, GO:0008270, GO:0016887, GO:0006508, GO:0030163, GO:0051301	-2.28
FP0609	Hypothetical protein	FP0609, FPSM_01724	GO:0008236, GO:0006508	2.71
FP0668	Probable ABC-type Na(+)-transporting system, permease component	*natB*, FPSM_01654	GO:0016021	2.26
FP0683	Transketolase, C-terminal subunit	*tktC*, FPSM_01638	GO:0004802, GO:0008152	-2.55
FP0698	Hypothetical protein	FPSM_01623, FP0698	GO:0016021	5.51
FP0699	Probable transmembrane protein	FP0699, FPSM_01622	GO:0016021, GO:0005524, GO:0016874, GO:0008152	3.54
FP0716	Probable transmembrane protein of unknown function	FP0716, FPSM_01604	GO:0016021	-3.72
FP0720	Aminopeptidase N	FP0720	GO:0004177, GO:0006508	3.51
FP0739	Ornithine--oxo-acid aminotransferase	*rocD*	GO:0004587, GO:0030170, GO:0008152	-2.31
FP0743	Glycosyltransferase	FP0743, FPSM_01574	GO:0016757, GO:0008152	2.41
FP0761	Acyltransferase	FPSM_01556, FP0761	GO:0016021, GO:0016747, GO:0008152	3.62

FP0853	ACP phosphodiesterase	FP0853, IA01_03995	GO:0008770, GO:0006633	2.33
FP0874	Threonine aldolase	IA01_04110, *ltaE*	GO:0004793, GO:0006520	3.08
FP0908	Succinyl-CoA synthetase, beta subunit	*sucC*	GO:0000287, GO:0004775, GO:0005524, GO:0030145, GO:0006099	-3.26
FP0921	Lipoprotein releasing system ATP-binding protein LolD	*lolD*, FP0921	GO:0043190, GO:0005524, GO:0016887, GO:0042954, GO:0008152, GO:0042953	3.29
FP0922	Lipoprotein releasing system transmembrane protein LolC	*lolC*, IA01_04335, FP0922	GO:0005886, GO:0016021	3.72
FP0936	Probable transmembrane protein	FP0936, FPSM_01336	GO:0016021	3.65
FP0938	Glycosyl hydrolase family 5	FP0938, FPSM_01338	GO:0016021, GO:0004553, GO:0005975	2.47
FP0939	Glycosyl transferase family 1	FP0939, FPSM_01339	GO:0016757, GO:0008152	2.36
FP0940	Sugar isomerase	FPSM_01340, FP0940	GO:0016021, GO:0016853, GO:0008152	4.77
FP0954	Imidazole glycerol phosphate synthase subunit HisF	*hisF*	GO:0005737, GO:0000107, GO:0016829, GO:0000105	4.00
FP0955	1-(5-phosphoribosyl)-5-[(5-phosphoribosylamino) methylideneamino] imidazole-4-carboxamide isomerase	*hisA*	GO:0005737, GO:0003949, GO:0000105	3.84
FP0958	Histidinol-phosphate transaminase	*hisC*	GO:0004400, GO:0030170, GO:0080130, GO:0000105	2.52
FP0960	ATP phosphoribosyltransferase	*hisG*	GO:0005737, GO:0000287, GO:0003879, GO:0005524, GO:0000105	2.97
FP0972	Succinyl-CoA synthetase, alpha subunit	FPSM_01372, *sucD*	GO:0004775, GO:0005524, GO:0048037, GO:0008152	-3.46
FP0974	UDP-3-O-(3-hydroxymyristoyl) glucosamine N-acyltransferase	FP0974, FPSM_01374	GO:0016747, GO:0009245	-2.54
FP0983	Probable TonB-dependent outer membrane receptor precursor	IA01_04635, FP0983	GO:0016020, GO:0004872, GO:0006810	2.67
FP0997	3-hydroxybutyryl- dehydrogenase	FP0997, SU65_04525	GO:0003857, GO:0008691, GO:0070403, GO:0006631, GO:0055114	-2.36
FP1025	Glutamate synthase	FPSM_01426, FP1025	GO:0016021, GO:0016041, GO:0006537, GO:0055114	3.35
FP1026	Gentisate 1,2-dioxygenase	IA01_04855, FP1026	GO:0016787, GO:0051213, GO:0055114	2.75

FP1037	Transglutaminase	FP1037, FPSM_01438	GO:0016021	2.97
FP1065	Mechanosensitive ion channel	*mscL*	GO:0005886, GO:0016021, GO:0005216, GO:0034220	-2.74
FP1093	Probable transmembrane protein of unknown function	FP1093, IA01_05185	GO:0016021	2.00
FP1130	Chromosome partitioning protein	FPSM_02042, *parB*	GO:0003677	-2.35
FP1155	Fatty acid desaturase	FP1155	GO:0045300, GO:0006631, GO:0055114	-2.32
FP1164	Aconitate hydratase	FPSM_02076, *acnA*	GO:0003994, GO:0051539, GO:0006099	-2.13
FP1199	Probable outer membrane protein precursor, Omp121 family	FP1199, FPSM_02109	GO:0016020, GO:0004872, GO:0006810	-3.39
FP1217	Glyceraldehyde-3-phosphate dehydrogenase	FPSM_02127, *gapA2*	GO:0004365, GO:0050661, GO:0051287, GO:0006006, GO:0055114	-3.76
FP1221	Alpha beta hydrolase	FPSM_02131	GO:0016787, GO:0008152	3.67
FP1228	Mechanosensitive ion channel	FPSM_02138, *mscS3*	GO:0016021, GO:0055085	2.09
FP1243	Probable peptidase	FPSM_02154, FP1243	GO:0016021, GO:0008236, GO:0006508	2.48
FP1245	Probable transmembrane protein of unknown function	FPSM_02156, FP1245	GO:0016021	3.00
FP1246	Alpha-1,4-N-acetylgalactosamine transferase	FPSM_02157, FP1246	GO:0016757, GO:0008152	2.22
FP1247	Glycosyl transferase	FPSM_02158, FP1247	GO:0016757, GO:0008152	2.91
FP1256	Sialic acid synthase (N-acetylneuraminic acid synthetase)	*neuB*	GO:0003824, GO:0016051	2.34
FP1263	Hypothetical protein	FP1263	GO:0016021, GO:0016747, GO:0008152	2.97
FP1264	Glycosyl transferase, group 2 family	FPSM_02175, FP1264	GO:0016757, GO:0008152	2.33
FP1308	Type II citrate synthase	*gltA*	GO:0005737, GO:0004108, GO:0006099	-2.58
FP1319	Pre-translocase subunit	*secY*	GO:0005622, GO:0005886, GO:0016021, GO:0006605, GO:0043952, GO:0065002	-2.65
FP1372	Glycine dehydrogenase	IA01_06590, *gcvP*	GO:0004375, GO:0006546, GO:0055114	-2.55
FP1384	Pyruvate dehydrogenase	FPSM_02299, *pdhC*	GO:0004742, GO:0008152	-2.09
FP1407	Fe-S oxidoreductase	IA01_06750, FP1407	GO:0051536, GO:0051989, GO:0055114	2.46

FP1409	Nucleoside diphosphate kinase	*ndk*	GO:0005737, GO:0004550, GO:0005524, GO:0046872, GO:0006165, GO:0006183, GO:0006228, GO:0006241	-2.10
FP1421	Prenyltransferase	*ubiA*, FP1421	GO:0016021, GO:0004659, GO:0008152	2.31
FP1429	Triose-phosphate isomerase	*tpiA*	GO:0005737, GO:0004807, GO:0006094, GO:0006096, GO:0006098	-3.02
FP1460	Bifunctional protein: cob(II)yrinic acid a,c-diamide reductase and nicotinate-nucleotide--dimethylbenzimidazole phosphoribosyltransferase	*bluB*/*cobT*	GO:0016021, GO:0008939, GO:0015420, GO:0016491, GO:0009163, GO:0009236, GO:0015889, GO:0035461, GO:0055114	4.55
FP1461	Bifunctional protein cobU: adenosylcobinamide kinase and adenosylcobinamide-phosphate guanylyltransferase	*cobU*, FP1461	GO:0005525, GO:0008820, GO:0043752, GO:0009236, GO:0016310, GO:0051188	3.52
FP1494	Hypothetical protein	FP1494, FPSM_01965	GO:0016021	-3.23
FP1512	Twin-arginine translocation pathway signal sequence domain precursor	FPSM_01986, FP1512	GO:0003824, GO:0008152	3.28
FP1606	Fumarate hydratase	*fumC*	GO:0045239, GO:0004333, GO:0006099, GO:0006106	-2.57
FP1609	Probable permease YojE	FPSM_01203, *yojE*, FP1609	GO:0016021	2.43
FP1610	Magnesium chelatase	FPSM_01202, FP1610	GO:0003677, GO:0005524, GO:0006260	3.09
FP1620	Putative transporting permease	FP1620, FPSM_01192	GO:0016021	2.78
FP1624	Probable lipoprotein precursor	FP1624, FPSM_01187	GO:0016021	-3.14
FP1626	tRNA(Ile)-lysidine synthase	*tilS*	GO:0005737, GO:0005524, GO:0016879, GO:0006400	2.09
FP1672	Probable transmembrane protein of unknown function Fjo11	*fjo11*, FPSM_01136	GO:0016021	2.63
FP1690	Potassium-transporting ATPase C subunit	*kdpC*	GO:0005887, GO:0005524, GO:0008556, GO:0008152, GO:0071805	3.18
FP1691	Potassium-transporting ATPase B subunit	*kdpB*	GO:0005887, GO:0000287, GO:0005524, GO:0008556, GO:0008152, GO:0071805	3.05

FP1692	Potassium-transporting ATPase A subunit	*kdpA*	GO:0005887, GO:0008556, GO:0030955, GO:0008152, GO:0071805	3.27
FP1699	Probable transmembrane protein of unknown function	FP1699	GO:0016021	-2.45
FP1703	Hypothetical protein	FP1703, IA01_08275	GO:0016021	2.56
FP1715	Major facilitator superfamily (MFS) permease	FP1715, FPSM_01091	GO:0016021	2.30
FP1741	Branched-chain amino acid aminotransferase	FPSM_01064, FP1741	GO:0016829, GO:0052654, GO:0052655, GO:0052656, GO:0009082	-2.65
FP1753	Peptide deformylase	*def*	GO:0005506, GO:0042586, GO:0006412	-2.69
FP1764	ABC transporter ATP-binding	FPSM_01042, FP1764	GO:0005524, GO:0016887, GO:0008152	-2.17
FP1773	Probable chloride channel protein	FPSM_01031, FP1773	GO:0016021, GO:0005247, GO:0006821, GO:0034220, GO:1903959	2.55
FP1784	Probable transmembrane protein of unknown function (membrane spanning)	FPSM_01018, FP1784	GO:0016021	3.52
FP1785	Probable TonB-dependent outer membrane receptor precursor	FPSM_01017, FP1785	GO:0009279, GO:0004872, GO:0006810	2.98
FP1800	Hypothetical protein	FP1800, FPSM_01002	GO:0016021	-2.35
FP1804	8-amino-7-oxononanoate synthase	FP1804	GO:0008890, GO:0016874, GO:0030170, GO:0009058	-2.40
FP1836	Dihydrodipicolinate synthase	*dapA*	GO:0005737, GO:0008840, GO:0009089, GO:0019877	-2.08
FP1842	Trans-2-enoyl- reductase	*fabV*, FP1842	GO:0004318, GO:0051287, GO:0006633, GO:0055114	-2.46
FP1845	Hypothetical protein (Membrane spanning	FPSM_00954, FP1845	GO:0016021	3.39
FP1865	Glycosyl transferase family 1	FPSM_00935, *wbsE*	GO:0016757, GO:0008152	3.00
FP1872	Isocitrate dehydrogenase	FP1872	GO:0000287, GO:0004450, GO:0051287, GO:0006099, GO:0006102	-2.57
FP1888	Probable M1 family aminopeptidase precursor	FP1888, FPSM_00912	GO:0004177, GO:0006508	-2.64
FP1890	Peptidylprolyl isomerase	FPSM_00910, *ppiB*	GO:0003755, GO:0000413, GO:0006457	-2.70

FP1930	Amino acid permease	FP1930	GO:0016021	2.76
FP1969	FAD-dependent oxidoreductase	*fjo30*	GO:0016491, GO:0055114	2.03
FP1989	Inward rectifier potassium channel Irk	FPSM_00803, *irk*	GO:0016021, GO:0005242, GO:0071805	2.32
FP2023	Inorganic polyphosphate kinase (NAD(+) kinase)	*ppnK*, *nadK*, FP2023	GO:0005737, GO:0003951, GO:0005524, GO:0046872, GO:0006741, GO:0016310, GO:0019674	2.19
FP2035	Probable transmembrane protein of unknown function	FP2035, IA01_10045	GO:0016021	5.78
FP2089	ABC transporter permease	IA01_10330, FP2089	GO:0016021	3.45
FP2098	Outer membrane protein precursor OmpH family P18	*ompH*, FP2098	GO:0051082	-4.18
FP2099	Glutamate racemase	*murI*	GO:0008881, GO:0008360, GO:0009252, GO:0071555	2.41
FP2100	Acetyltransferase	FP2100	GO:0016740, GO:0008152	3.27
FP2119	Teicoplanin resistance (Probable transmembrane protein of unknown function Fjo27)	*fjo27*	GO:0016021	-2.81
FP2120	Glycine cleavage system H	*gcvH*	GO:0005960, GO:0019464	-2.97
FP2133	Protein of unknown function containing peptidoglycan binding domain	FPSM_00652, FP2133	GO:0016740, GO:0008152	2.08
FP2195	Probable ABC-type transport system, permease component	FP2195, FPSM_02307	GO:0005886, GO:0016021	3.64
FP2209	Biopolymer transporter (Biopolymer transport ExbD1 protein)	*exbD1*	GO:0005886, GO:0016021, GO:0005215, GO:0015031	-2.61
FP2234	Membrane carboxypeptidase/penicillin-binding protein PbpC	*pbpC*, IA01_11080, FP2234	GO:0016021, GO:0004180, GO:0008658, GO:0008955, GO:0006508, GO:0009252	2.14
FP2254	Acyl- :6-aminopenicillanic acid acyl-transferase	IA01_11180, FP2254	GO:0016740, GO:0016811, GO:0008152	2.33
FP2256	Glycerol acyltransferase	FP2256	GO:0016021, GO:0003841, GO:0008152	2.54
FP2260	Hypothetical protein	IA01_11210	GO:0016021	-2.54

FP2271	ABC-type transport system, permease component	FP2271	GO:0016021, GO:0006810	2.00
FP2284	3-oxoacyl-(acyl-carrier-protein) synthase I	*fabB*, FPSM_02405	GO:0004315, GO:0008152	-2.27
FP2337	Amino acid transporter	FP2337, IA01_11610	GO:0016021, GO:0015197, GO:0006857	2.15
FP2362	Probable membrane-associated phospholipid phosphatase	FP2362	GO:0016020	3.01
FP2369	Aminopeptidase	IA01_11780, *pepC*, FP2369	GO:0004177, GO:0004197, GO:0006508	-2.56
FP2375	Phosphoenolpyruvate carboxykinase	*pckA*	GO:0005737, GO:0004612, GO:0005524, GO:0016301, GO:0046872, GO:0006094, GO:0016310	-2.50
FP2377	Acetyl-CoA carboxylase biotin carboxylase subunit	FPSM_02506, *accC*	GO:0003989, GO:0004075, GO:0005524, GO:0046872, GO:0008152	-2.42
FP2381	DNA-binding	IA01_11840	GO:0003677	-2.20
FP2392	Cysteine desulfurase	*sufC*	GO:0005524, GO:0016887, GO:0006810, GO:0008152	-2.11
FP2409	2-oxoglutarate dehydrogenase (Dihydrolipoyllysine-residue succinyltransferase component of 2-oxoglutarate dehydrogenase complex)	*sucB*, FPSM_02538	GO:0045252, GO:0004149, GO:0006099, GO:0033512	-2.30
FP2416	Bifunctional purine biosynthesis protein PurH	*purH*	GO:0003937, GO:0004643, GO:0006189	-2.23
FP2417	Rod shape-determining protein MreB	*mreB*, FPSM_02546, FP2417	GO:0000902	-2.58
FP2434	Pantothenate kinase (Putative transcriptional regulator=)	*coaX*, FP2434	GO:0005737, GO:0004594, GO:0005524, GO:0046872, GO:0015937, GO:0016310	2.11
FP2446	Addiction module toxin, Txe/YoeB family (endonuclease activity)	FP2446	GO:0004519, GO:0006401, GO:0090305	-2.67
FP2484	Pre-translocase subunit	FP2484	GO:0005887, GO:0033281, GO:0008320, GO:0009306, GO:0043953	-2.15
FP2496	Probable transmembrane protein of unknown function	FPSM_02631, FP2496	GO:0016021	-2.71

FP2581	Hypothetical protein	FPSM_02286, FP2581	GO:0016021	3.20
FP2614	Probable biotin--[acetyl-CoA-carboxylase] ligase	*birA*, IA01_03035, FP2614	GO:0004077, GO:0006464	2.80
FP0017	Putative cell surface protein precursor SprF	FP0017		-2.83
FP0056	Hypothetical protein	FP0056		-2.11
FP0091	Hypothetical protein	FP0091		4.20
FP0134	Hypothetical protein	FP0134		-2.70
FP0135	Hypothetical protein	FP0135		-2.46
FP0212	Hypothetical protein	FP0212		3.78
FP0266	Hypothetical protein (GLPGLI family)	FP0266		2.43
FP0383	Hypothetical protein (membrane associated)	FP0383		-5.71
FP0396	Hypothetical protein	FP0396		-3.71
FP0407	Hypothetical lipoprotein precursor	FP0407		-3.34
FP0417	Hypothetical protein	FP0417		2.05
FP0440	Probable outer membrane protein precursor	FP0440		2.72
FP0451	Hypothetical protein	FP0451		-2.81
FP0459	Hypothetical protein FPSM_00504	FP0459		2.13
FP0466	Fructose 1,6-bisphosphatase	FP0466		-2.04
FP0535	Probable lipoprotein precursor	FP0535		-2.02
FP0550	von Willebrand factor A (Probable outer membrane protein precursor YfbK)	*yfbK*, FP0550		2.34
FP0567	Hypothetical protein	FP0567		2.61
FP0617	Hypothetical protein (membrane spanning)	FP0617		4.29
FP0620	Hypothetical protein (possible oxidoreductase)	FP0620		2.55
FP0700	Hypothetical protein	FP0700		2.88

FP0705	Hypothetical protein	FP0705	4.61
FP0738	Hypothetical protein	FP0738	2.80
FP0852	Hypothetical protein (membrane)	FP0852	-2.85
FP0906	Hypothetical protein (DUF1456 domain containing protein)	FP0906	-2.97
FP0928	Thiol: disulfide interchange lipoprotein precursor, N-terminal region	FP0928	-2.21
FP0937	Hypothetical protein	FP0937	3.25
FP0985	Hypothetical protein	FP0985	2.14
FP1057	Hypothetical protein	FP1057	-2.21
FP1113	Asparagine synthetase B (Protein of unknown function precursor)	FP1113	2.31
FP1151	DUF2805 domain containing	FP1151	-4.43
FP1198	Probable lipoprotein precursor	FP1198	-3.63
FP1212	Hypothetical protein	FP1212	2.29
FP1344	Probable outer membrane protein precursor	FP1344	2.50
FP1431	Hypothetical protein	FP1431	-2.98
FP1456	Probable lipoprotein precursor	FP1456	-2.87
FP1462	Hypothetical protein	FP1462	3.50
FP1478	Probable lipoprotein precursor	FP1478	-2.80
FP1487	Iron-regulated protein A precursor	FP1487	-3.83
FP1493	Probable lipo precursor	FP1493	-4.42
FP1508	Probable lipoprotein precursor	FP1508	-2.74
FP1511	Hypothetical protein	FP1511	2.61
FP1652	Hypothetical protein (membrane spanning)	FP1652	-2.41
FP1675	Hypothetical protein	FP1675	-2.27
FP1689	Protein of unknown function precursor (outer membrane)	FP1689	2.58

FP1710	Hypothetical protein (primase)	FP1710	-2.64
FP1737	Hypothetical protein, partial	FP1737	2.61
FP1754	Hypothetical protein	FP1754	-3.17
FP1770	Protein of unknown function containing tetratricopeptide repeats	FP1770	-2.09
FP1788	Hypothetical protein	FP1788	-3.32
FP1837	Outer membrane assembly lipoprotein precursor BamD (M: Cell wall/membrane/envelope biogenesis)	FP1837	-2.67
FP2005	Hypothetical protein (SRPBCC superfamily)	FP2005	-2.10
FP2017	Putative outer membrane protein precursor	FP2017	2.67
FP2073	Hypothetical protein	FP2073	3.87
FP2205	Tetratricopeptide repeat family	FP2205	-3.05
FP2351	Hypothetical protein	FP2351	-2.35
FP2361	Hypothetical protein	FP2361	2.30
FP2384	Protein of unknown function precursor	FP2384	-2.52
FP2424	Probable lipoprotein precursor	FP2424	-2.89
FP2425	Probable lipoprotein precursor	FP2425	-2.14
FP2463	RCC1 (Regulator of Chromosome Condensation) repeat domain precursor	FP2463	2.22
FP2466	Hypothetical protein	FP2466	-2.00

(*) Hit sequences based on BLAST algorithm run against the non-redundant (NR) database at NCBI.

1.4 Supplementary Table 4

Table S4. *F. psychrophilum*-induced cytotoxicity modeling in GAMLSS using different predictor variables. Pr(Chi) values in bold indicate predictor variables that were considered statistically significant.

Model	Strain considered in the model	Predictor variable	LRT	Pr(Chi)	R-squared (%)
Model 1	LM-02-Fp and NCMB1947^T	Growth state	8.7672	**0.0030669**	18.16
		Time post-infection	14.1727	**0.0001668**	
		Strain	2.49	0.1145734	
Model 2	LM-02-Fp	Growth state	1.5739	0.209637	19.29
		Time post-infection	11.6099	**0.000656**	
		Interaction between growth state and time post-infection	0.52913	0.467	
Model 3	NCMB1947^T	Growth state	12.2558	**0.0004638**	27.99
		Time post-infection	3.0872	0.078912	
		Interaction between growth state and time post-infection	3.6123	**0.05736**	
