# Supplementary figures and images for: Stress Tolerance-Related Genetic Traits of Fish Pathogen Flavobacterium psychrophilum in a Mature Biofilm
Source: Front Microbiol. 2018 Jan 23;9:18. doi: 10.3389/fmicb.2018.00018 (PMC5787105; doi:10.3389/fmicb.2018.00018)

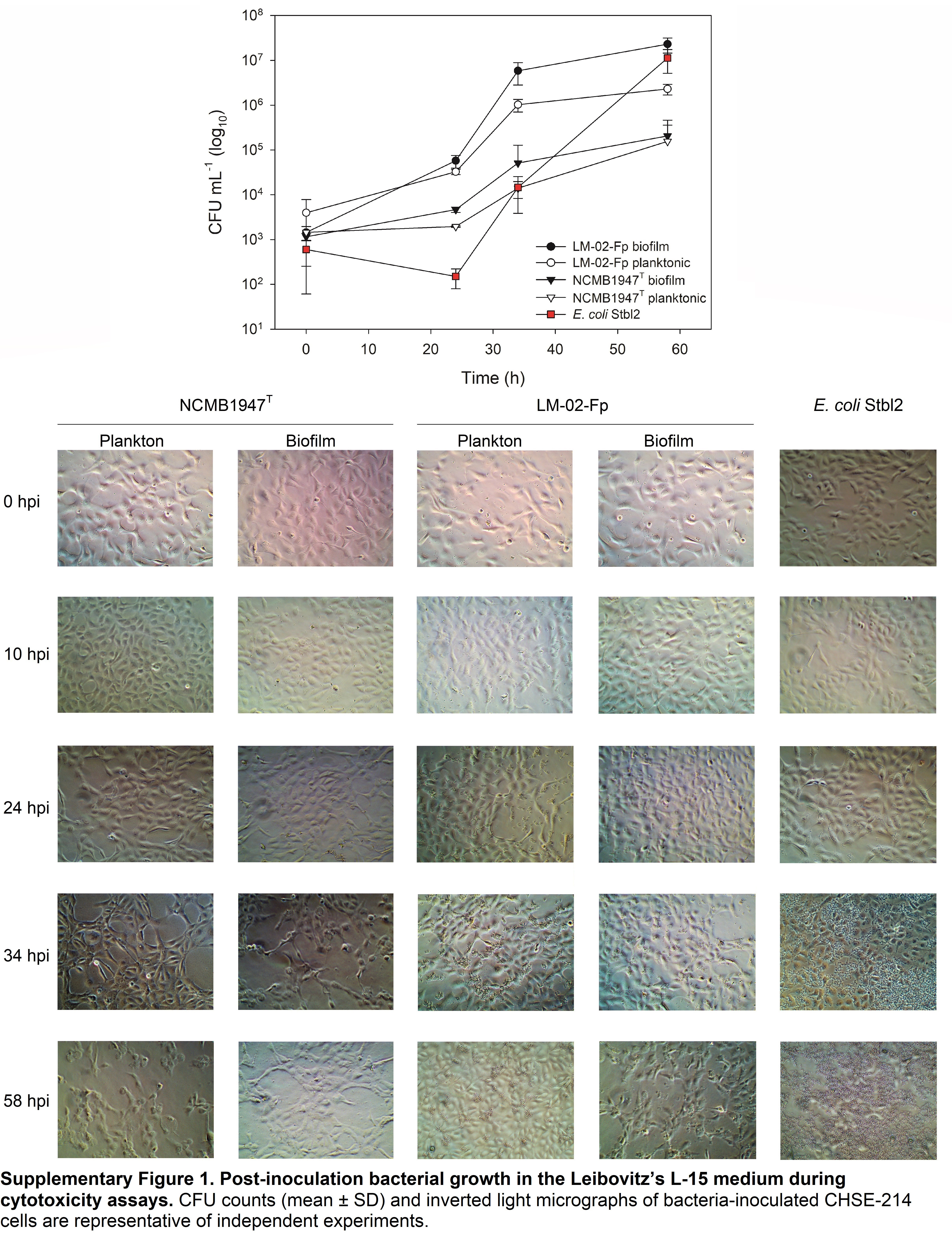

Supplement: Supplementary file 2 [file Image1.JPEG]
